# Injectable and Near-Infrared Light-Controllable Fibrin Hydrogels with Antimicrobial and Immunomodulating Properties for Infected Wound Healing

**DOI:** 10.34133/bmr.0019

**Published:** 2024-06-27

**Authors:** Qing Zhang, Yongxian Jiang, Xiaolong Zhang, Yi Wang, Rong Ju, Guoqing Wei

**Affiliations:** ^1^ School of Life Science and Engineering, Southwest Jiaotong University, Chengdu 610031, China.; ^2^ Chengdu Women’s and Children’s Central Hospital, School of Medicine, University of Electronic Science and Technology of China, Chengdu 611731, China.; ^3^ Sichuan Provincial Maternity and Child Health Care Hospital, the Affiliated Women’s and Children’s Hospital of Chengdu Medical College, Chengdu 610041, China.

## Abstract

The management of infected wounds poses a significant challenge due to the growing issue of antibiotic resistance, underscoring the urgent necessity to innovate and implement alternative therapeutic strategies. These strategies should be capable of eliminating bacterial infections in infected wounds while circumventing the induction of multi-drug resistance. In the current study, we developed an easily prepared and injectable fibrin gel (FG) loaded with nanoparticles (NPs) that exhibit antibacterial and immunomodulatory properties to facilitate the healing of infected wounds. Initially, a novel type of NP was generated through the electrostatic interaction between the photothermal agent, mPEG-modified polydopamine (MPDA), and the nitric oxide (NO) donor, S-nitrosocysteamine (SNO). This interaction resulted in the formation of NPs referred to as SNO-loaded MPDA (SMPDA). Subsequently, the SMPDA was encapsulated into the FG using a double-barreled syringe, thereby producing the SMPDA-loaded FG (SMPDA/G). Experimental results revealed that SMPDA/G could effectively eliminate bacterial infections and alter the immune microenvironment. This efficacy is attributed to the synergistic combination of NO therapy and photothermal therapy, along with the role of SMPDA in facilitating M2 macrophage polarization within the gel. Accordingly, these findings suggest that the SMPDA/G holds substantial promise for clinical application in infected wound healing.

## Introduction

Burns, trauma, surgical procedures, and chronic conditions such as diabetes are known to disrupt the structural integrity and defensive role of human skin. This disruption makes the skin more vulnerable to various bacterial pathogens, leading to wound infections. Such infections are associated with delayed wound healing (WH) and considerable economic costs and represent a substantial threat to public health globally [1–3]. In recent decades, while conventional antibiotic therapies have been the primary approach for combating bacterial infection (BI), their misuse, particularly the excessive application of antibiotics, has contributed to the rise and expansive distribution of a variety of multidrug-resistant (MDR) bacteria. This development has critically weakened the effectiveness of antibiotic therapies [4,5]. Alarmingly, bacteria are estimated to evolve resistance to antibiotics in a period shorter than 2 weeks. This rapid emergence of resistance substantially exceeds the rate at which new antibiotics are discovered [6]. The escalation of bacterial resistance is turning into a critical and potentially lethal issue on a global scale, accounting for approximately 7 million fatalities annually [6]. Consequently, the increasing difficulty posed by antibiotic resistance significantly complicates the management of infected wounds [7]. Therefore, it is critically important and has become an urgent priority to devise strong alternative therapeutic strategies to effectively combat BI while avoiding the induction of MDR [8,9].

Moreover, the development of bacterial biofilms is a significant contributor to persistent infections, severely hindering the healing of wounds. The unique composition and organization of these biofilms provide a natural defense against the infiltration of antibacterial agents, resulting in increased antibiotic tolerance and resistance [10,11]. Nitric oxide (NO), a type of free radical endogenously produced in the body, serves as a crucial regulator in numerous physiological and pathological processes, including immunological responses and WH [12]. The antibacterial properties of NO are attributed to its capacity to cause nitrosative or oxidative stress on bacterial membranes. This stress mechanism involves lipid peroxidation and DNA deamination, enabling NO to exhibit extensive antibacterial activity, especially against bacteria that have developed resistance [13]. The ultralow molecular weight of NO facilitates its effective diffusion through biological membranes without the necessity of any active transport mechanisms [14]. Furthermore, multiple studies have highlighted that NO could trigger the dispersion of biofilms in various bacterial species, including *Pseudomonas aeruginosa* [15], *Staphylococcus aureus* [16], and other bacterial species [17,18]. The effectiveness of NO may be attributed to its ability to lower cyclic diguanylate concentrations, thereby repressing bacteria-surface attachment and biofilm formation [19,20]. Therefore, NO emerges as a promising non-antibiotic strategy for the treatment of infected wounds. Nevertheless, the use of NO in gas therapy is constrained by its limited half-life [21]. This limitation underscores the need to engineer various NO donors capable of releasing NO in response to specific microenvironmental conditions of diseases (such as pH, enzymes, and glutathione) or in reaction to external stimuli (like light or ultrasound).

Recent advancements have seen the introduction of several cutting-edge and effective techniques, such as photodynamic therapy, photothermal therapy (PTT), chemodynamic therapy, and sonodynamic therapy, for the elimination of BI [22]. Among these antimicrobial methods, antibacterial PTT (aPTT) has attracted increasing attention due to its array of significant benefits, such as precise control, reduced adverse effects, minimal invasiveness, rapid and effective antibacterial action, broad-spectrum activity against bacteria, and low risk of bacterial resistance development [23,24]. The aPTT has been recognized for minimal invasiveness based on photochemical mechanisms to counteract drug resistance. It converts light energy, often from the near-infrared (NIR) spectrum, into heat through photothermal agents (PTAs) when irradiated at targeted areas. This heat is responsible for bacterial cell destruction by impairing bacterial cell membranes and triggering protein denaturation [24]. Extensive research has focused on the utilization of diverse inorganic substances, such as gold nanoparticles (NPs), carbon-based nanomaterials, and organic NPs (e.g., porphyrins, polyaniline, and polydopamine [PDA]) for their inherent photothermal abilities in bacterial elimination [25]. Notably, a range of materials with the capacity for photothermal conversion have been innovatively designed to deliver other therapeutic substances, including antibiotics and gas donor molecules, directly to infected areas. This strategy is intended to enhance antimicrobial treatment efficacy under NIR light exposure [26,27]. It is also important to acknowledge that the overactivation of M1 macrophages and the subsequent secretion of pro-inflammatory factors often lead to chronic, non-healing wounds [28,29]. Thus, modulating the microenvironment immunologically presents a viable approach for the healing of chronic wounds. In this context, PDA, a prominent natural analog of melanin, emerges as a highly promising PTA due to its simple fabrication process, remarkable photostability, beneficial biodegradability, and excellent biocompatibility [30]. To date, numerous investigations have substantiated that PDA serves not only as an antibacterial PTA but also as an immunomodulator, facilitating the polarization of M2 macrophages to accelerate WH [31]. Given these insights, we hypothesized that the development of an integrated system, encompassing immunomodulation, aPTT, and NO therapy, holds promise for concurrently addressing multiple targets, thereby prominently enhancing the synergistic effect on WH promotion. Nonetheless, such a system remains to be established.

**Fig. 1.**
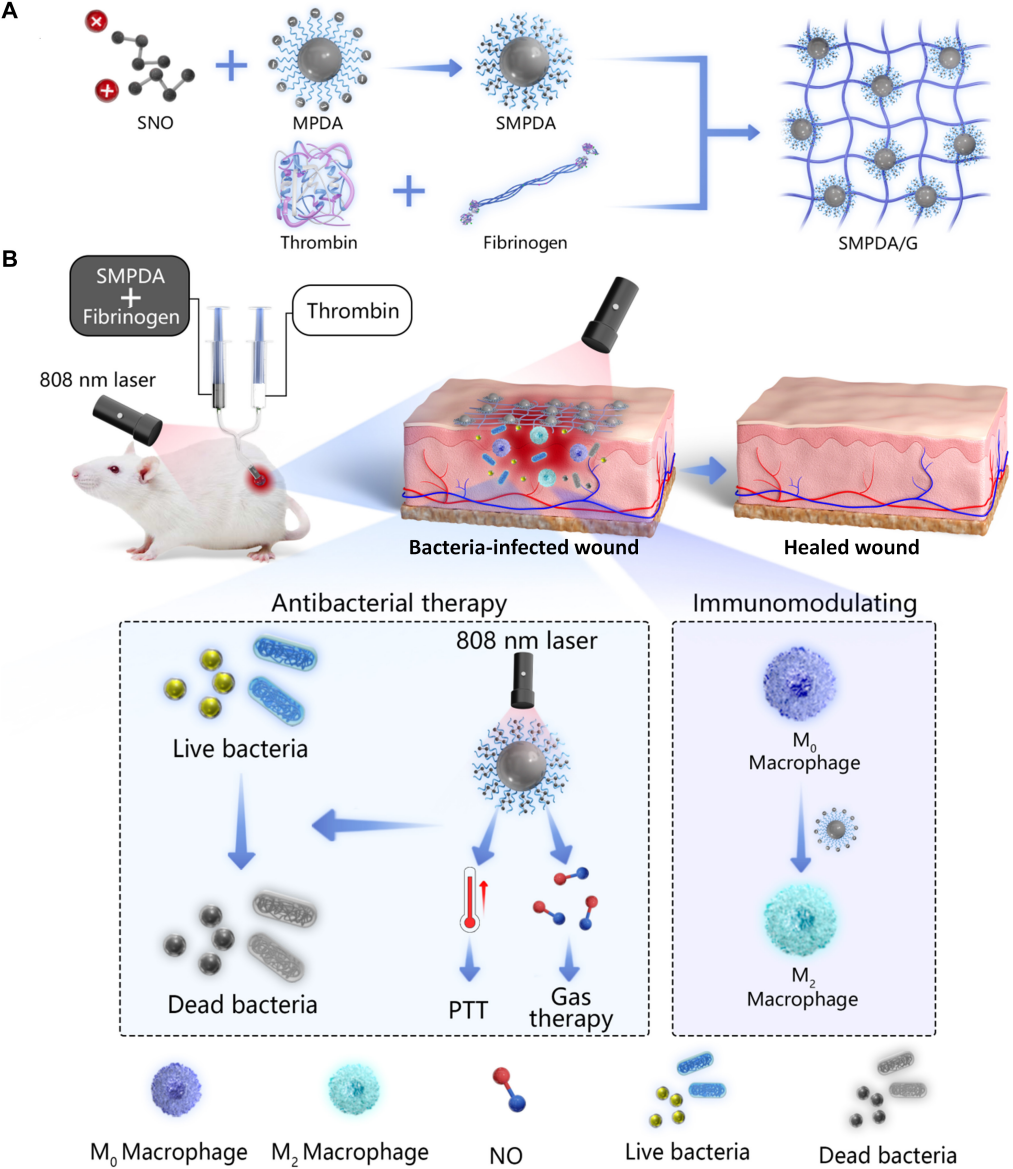
Schematic illustration of the preparation of (A) SMPDA and SMPDA/G fibrin gel, and (B) therapeutic mechanism of SMPDA/G fibrin gel for infected wound healing.

This study aimed to devise an injectable NP-loaded fibrin gel (FG) that can be easily prepared and exhibits a wide array of antibacterial and immunomodulatory characteristics for addressing infected wounds. Notably, the FG used in this study is an approved material by the US Food and Drug Administration and is formed through the interaction between fibrinogen and thrombin [32]. In this study, we capitalized on the distinctive advantages of FG, including exceptional biocompatibility, a user-friendly injectable delivery method for WH, and the ability to enhance WH by establishing a temporary barrier that connected and shielded injured tissue. Initially, a novel type of NPs was successfully synthesized through the electrostatic interaction between the PTA mPEG-modified PDA (MPDA) and the NO donor S-nitrosocysteamine (SNO), which was denoted as SNO-loaded MPDA (SMPDA). Subsequently, the SMPDA was incorporated into the FG to form SMPDA-loaded FG, referred to as SMPDA/G (Fig. 1A). Our findings confirmed that SMPDA NPs demonstrated exceptional photothermal properties and the capabilities to rapidly release NO due to increase in temperature upon exposure to 808-nm laser irradiation. This property facilitated a synergistic antimicrobial effect by combining PTT and NO therapy. Additionally, it should be highlighted that MPDA could stimulate the activation of M2-phenotype macrophages to orchestrate the immune microenvironment (IME) and consequently expedite WH (Fig. 1B). Overall, the SMPDA/G system displayed favorable biocompatibility and exhibited remarkable antimicrobial and immunomodulatory characteristics, thereby establishing it as a promising strategy for addressing infected wounds.

## Materials and Methods

### Materials

The materials employed in this study included an array of substances and reagents. A pivotal constituent, mPEG-NH_2_, characterized by a molecular weight of 2 kDa, was sourced from Ponsur Biotechnology (Shanghai, China). The procurement process further entailed the acquisition of substances including t-butyl nitrite, 1-(3-dimethylaminopropyl)-3-ethylcarbodiimide, N-hydroxysuccinimide, dialysis bags, and the Griess reagent from Aladdin Reagents Company (Shanghai, China). Dulbecco’s modified Eagle’s medium (DMEM) was acquired from Gibco (USA), while the Calcein-AM/propidium iodide (PI) cell viability/cytotoxicity assay and Cell Counting Kit-8 (CCK-8) assay kits were obtained from Beyotime in China. Additionally, phosphate buffer saline (PBS) and fetal bovine serum (FBS) were respectively obtained from Boster and HYCEZMBIO in China. Unless explicitly specified otherwise, all other chemicals adhered to analytical grade standards and were supplied by Changzheng Regents Company (Chengdu, China).

### Preparation and characterization of NPs

The production of MPDA NPs was undertaken following an established methodology [33], albeit with minor adjustments. The synthesis of the PDA NPs was conducted with a mixture of 10 ml of ammonia aqueous solution (28% to 30%), 120 ml of ethanol, and 270 ml of deionized water (DW). The amalgamated solution was subjected to mild magnetic stirring, with the entire procedure conducted at room temperature. DW (30 ml) containing 1.5 g of dopamine hydrochloride was added to the solution described earlier. Following a 12-h incubation period, the resulting product was isolated through a 30-min centrifugation at 12,000 rpm. Subsequently, it underwent 3 washes in DW. The quantification of PDA was carried out by measuring its weight following the process of lyophilization. In the synthesis of MPDA NPs, a suspension of PDA NPs and mPEG-NH_2_ was prepared in Tris–HCl buffer (pH 8.5, 10 mM) with a mass ratio of 1:2. This suspension was subjected to vigorous stirring for 24 h, following which the MPDA was separated via centrifugation.

The preparation of SMPDA involved the entrapment of SNO into MPDA using electrostatic adsorption. Briefly, SNO was synthesized by combining cysteamine hydrochloride (11.3 mg, 0.1 mmol) and t-butyl nitrite (11.3 mg, 0.11 mmol) in 1 ml of DW. This reaction was carried out at 0 °C in a light-restricted environment for a duration of 30 min [34]. Following the elimination of any remaining t-butyl nitrite via vacuum evaporation, a 1.0-ml solution of SNO (1.0 × 10^−3^ m) was allowed to react with 1.0 mg of MPDA for a duration of 4 h at 0 °C without light exposure. Afterward, the SMPDA was isolated by centrifugation (at 12,000 rpm) and underwent several rounds of washing with water before being collected through additional centrifugation.

The morphology and size of the 3 aforementioned NPs were subject to examination via transmission electron microscopy (TEM) (JEM-2100 Plus, JEOL, Japan). Furthermore, the size and surface charge of NPs were examined using dynamic light scattering (DLS) on a Zeta-sizer, Nano-ZS90 (Malvern Instruments, UK).

### Formation and characterization of NP-loaded FGs

The formation of FGs was accomplished through a double-barreled syringe, whereby fibrinogen PBS solution (20 mg/ml) and thrombin PBS solution (100 U/ml) were mixed in various weight-to-unit (weight/U) ratios, specifically 2:1, 3:2, 1:1, 2:3, and 1:2. The preparation of NP-loaded FGs followed the same methodology mentioned earlier. Fibrinogen solution (20 mg/ml), incorporating MPDA or SMPDA, was combined with thrombin solution (100 U/ml) at a 3:1 (weight/U) ratio. The morphology of the resulting fibrin was subjected to characterization using a scanning electron microscope (SEM) (Sigma300, ZEISS, Germany).

### Swelling and degradation tests

To determine the swelling behavior, the pre-weighed freeze-dried G or SMPDA/G specimen (*W*_0_) was incubated in PBS at 37 °C until the equilibrium state was achieved. Then, the specimen was taken out at 10, 20, 40, 60, 120,180, 240, 300, 360, 420, 480, 540, and 600 s during the swollen process, residual liquids were removed on the surface of the specimen by using wet filter papers, and the wet weights (*W*_t_) of specimen were recorded. The swelling ratio was defined as: Swelling ratio = (*W*_t_ − *W*_0_)/*W*_0_.

To assess the degradation characteristics of the SMPDA/G, the specimen was first prepared and retained on a plastic plate. At a predetermined time (0, 0.5, 1, 2, and 3 days), the morphology change of the hydrogels was photographed.

### In vitro photothermal property of SMPDA and SMPDA/G

To evaluate the photothermal characteristics, SMPDA suspensions at concentrations of 50, 100, 200, and 400 μg/ml in PBS were positioned in 1.5-ml Eppendorf tubes. Additionally, SMPDA/G was prepared in a 12-well plate. These samples underwent continuous irradiation utilizing an 808-nm laser (Blueprint, Beijing, China) at various power levels (0.5 W/cm^2^, 1.0 W/cm^2^, and 1.5 W/cm^2^) for a duration of 10 min. The control groups in this study included pure FGs (G) and PBS. The photothermal stability of SMPDA was evaluated following laser irradiation. Specifically, SMPDA at a concentration of 200 μg/ml in PBS underwent irradiation with an 808-nm laser for 4 consecutive on/off cycles. Thermal data during this process were recorded using a Fluke Ti480 thermal camera.

### NO releasing test

NO releasing from the SMPDA and SMPDA/G were detected by the Griess Reagent method [35], respectively. In brief, 200 μg of SMPDA was dispersed in 1.0 ml of PBS buffer on the tube or 1.0 ml of SMPDA/G hydrogel formed was immersed in 1 ml of PBS buffer on the tube. After different treatments (continuous NIR irradiation, intermittent NIR irradiation, or without irradiation), 100 μl of the solution was removed to a 96-well plate and then 50 μl of Griess Reagent I and 50 μl of Griess Reagent II were added to the well plate and incubated for 5 min. The optical density (OD) value of mixed solution was read by a microplate reader at 540 nm. The amount of NO release was calculated from a standard curve established using NaNO_2_ solutions with known concentrations.

### Cell lines

Cell lines, including human umbilical vein endothelial cells (HUVECs), mouse fibroblasts (3T3), and RAW 246.7 macrophages, were cultured in DMEM with high glucose content, supplemented with 10% FBS. These cultures were maintained under a controlled environment with 5% CO_2_ at a temperature of 37 °C.

### In vitro and ex vivo biocompatibility evaluation

For in vitro cytotoxicity assay, HUVECs and 3T3 cells were plated in 96-well plates and allowed to undergo a 24-h incubation period. Thereafter, varying concentrations of SMPDA were introduced into the co-culture system for a specified duration. The cytotoxic effects were determined through the utilization of the CCK-8 assay. For ex vivo biocompatibility evaluation, the hemocompatibility assessment was conducted in accordance with established protocols [36].

### In vitro immunomodulating ability

RAW 264.7 macrophages, plated on 6-well plates, underwent treatment with 200 μg/ml SMPDA or remained untreated, in conjunction with exposure to 200 ng/ml lipopolysaccharide for a duration of 24 h. Subsequently, these RAW 264.7 cells were subjected to staining with anti-CD86 or anti-CD206 antibodies, with visualization facilitated by fluorescence microscopy. Furthermore, the quantification of the levels of IL-6, TNF-𝛼, and IL-10 in the supernatant of the cell culture was performed utilizing enzyme-linked immunosorbent assay (ELISA) kits [37,38].

### In vitro antimicrobial activity

The colony formation assay, adhering to the protocols established in our prior research [39], was undertaken to assess the *in vitro* antimicrobial properties of the biomaterials. Additionally, we sought to explore their photothermal antimicrobial capabilities by introducing MPDA and SMPDA into a bacterial suspension. These samples were subsequently subjected to irradiation using an 808-nm laser at 1.5 W/cm^2^ for 5 min, while a control group remained unexposed to laser radiation. Following a 24-h incubation period, the bacterial cultures underwent dilution in PBS. A 100-μl aliquot of the resulting suspension was applied to LB agar plates, after which the plates were subjected to a 24-h incubation at 37 °C. The resultant colony formation was documented through photography.

In the live/dead bacterial staining assay, a specific bacterial concentration was subjected to staining using PI dye and SYTO 9 Green Fluorescent at 37 °C for a duration of 20 min. Following this staining process, 10 μl of the stained bacterial sample was deposited onto a microscope slide, and images were captured utilizing a fluorescence microscope.

For the biofilm formation experiment, a bacterial concentration of 5 × 10^6^ CFU/ml of *S. aureus* was co-cultured with SMPDA in different concentrations. After exposure to an 808-nm laser at an intensity of 1.5 W/cm^2^ for 5 min, the bacterial suspension was distributed into a 96-well plate (200 μl per well) and incubated for 48 h at 37 °C. The incubated wells were washed lightly with PBS. After fixing with 4% paraformaldehyde, the bacterial biofilm was stained with 0.5% crystal violet for 10 min, then washed 3 times with PBS. The stained bacterial biofilm was treated with pure alcohol, then the absorbance at 590 nm was quantified.

### Mouse model of infected wounds

BALB/c (male, 4 to 6 weeks old) mice were used as the model of infected wound. The 1-cm full-thickness round skin wound was made on the backs of BALB/c mice. Then, 20 μl of *S. aureus* suspension (1 × 10^8^ CFU/ml) was dropped into the wound. The mice were randomly divided into 6 groups (*n* = 6): PBS group, G group, MPDA/G group, MPDA/G with NIR irradiation (MPDA/G+L) group, SMPDA/G group, and SMPDA/G with NIR irradiation (SMPDA/G+L) group, NIR (808 nm laser at 1.5 W/cm^2^ for 5 min), respectively. The photothermal images in all groups were recorded using a Fluke Ti480 thermal camera. The treatments were repeated every 2 days. The wounds were captured on days 0, 3, 7, and 12. The wound area was assessed using ImageJ software.

### In vivo biosafety evaluation

After treatment, the mice were sacrificed and the primary organs were obtained. The organs of mice were fixed and then stained with H&E to observe the pathological changes.

### In vivo antimicrobial effect of G

We obtained the scab from the infected wounds on day 6. The mixture was diluted with PBS and incubated on LB agar for 24 h. The colony formation was captured.

### Histological analysis

We obtained the wound bed tissues of the infected wound on day 12. H&E and Masson staining were performed [40]. Then, immunofluorescent staining analysis was performed to assess the expression of CD206, iNOS, and CD31, which were then quantified using ImageJ.

### Statistical analysis

The data were presented as mean ± standard deviation (SD) based on at least 3 tests. Comparisons were performed using Student’s *t* test between 2 groups or using one-way analysis of variance among multiple groups. Significant difference was defined as *P < 0.05 and **P < 0.01.

## Results and Discussion

### Preparation and characterization of NPs

PDA and MPDA were synthesized following established procedures with slight adjustments [41]. The chemical alteration of PDA was substantiated through Fourier transform infrared (FT-IR) spectra analysis (as depicted in Fig. S1), where distinct absorption bands at 1,090 cm^−1^ indicated the stretching vibration associated with -CH_2_–O–CH_2_- in mPEG. These FT-IR data affirmed the successful grafting of mPEG onto the surface of PDA. Furthermore, the determination of the polymer-grafting ratio of mPEG was conducted using thermal gravimetric analysis (TGA). As illustrated in Fig. S2, the weight percentage of unmodified PDA was calculated to be 66.3%. Upon functionalization with mPEG, the weight percentage of unmodified MPDA was calculated to be 58.0%. The observed rise in weight percentage was attributable to the inclusion of mPEG. These TGA curves provided additional evidence affirming the successful synthesis of MPDA. Based on the aforementioned findings, it can be ascertained that approximately 8.3% of mPEG has been grafted onto the material. To date, the most extensively studied NO donors include S-nitrosothiols, N-diazeniumdiolates, and organic nitrates. N-diazeniumdiolates have the capability to spontaneously produce NO under physiological conditions, while the SNO bond in S-nitrosothiols can be disrupted through exposure to heat and light irradiation [41]. In the current study, we achieved the successful synthesis of cationic and thermosensitive cationic and thermosensitive SNO through a well-established procedure involving the reaction between cysteamine and t-butyl nitrite, as detailed in prior research [42]. This synthesis was further substantiated by the identification of the characteristic absorption peak at 545 nm in the ultraviolet–visible spectra (Fig. S3). Due to the abundant presence of hydroxy groups on the surface of MPDA, an electrostatic adsorption technique was employed to graft SNO onto the MPDA surface, ultimately resulting in the formation of SMPDA, and the loading capacity of SNO in MPDA NPs was about 4.2%.

**Fig. 2.**
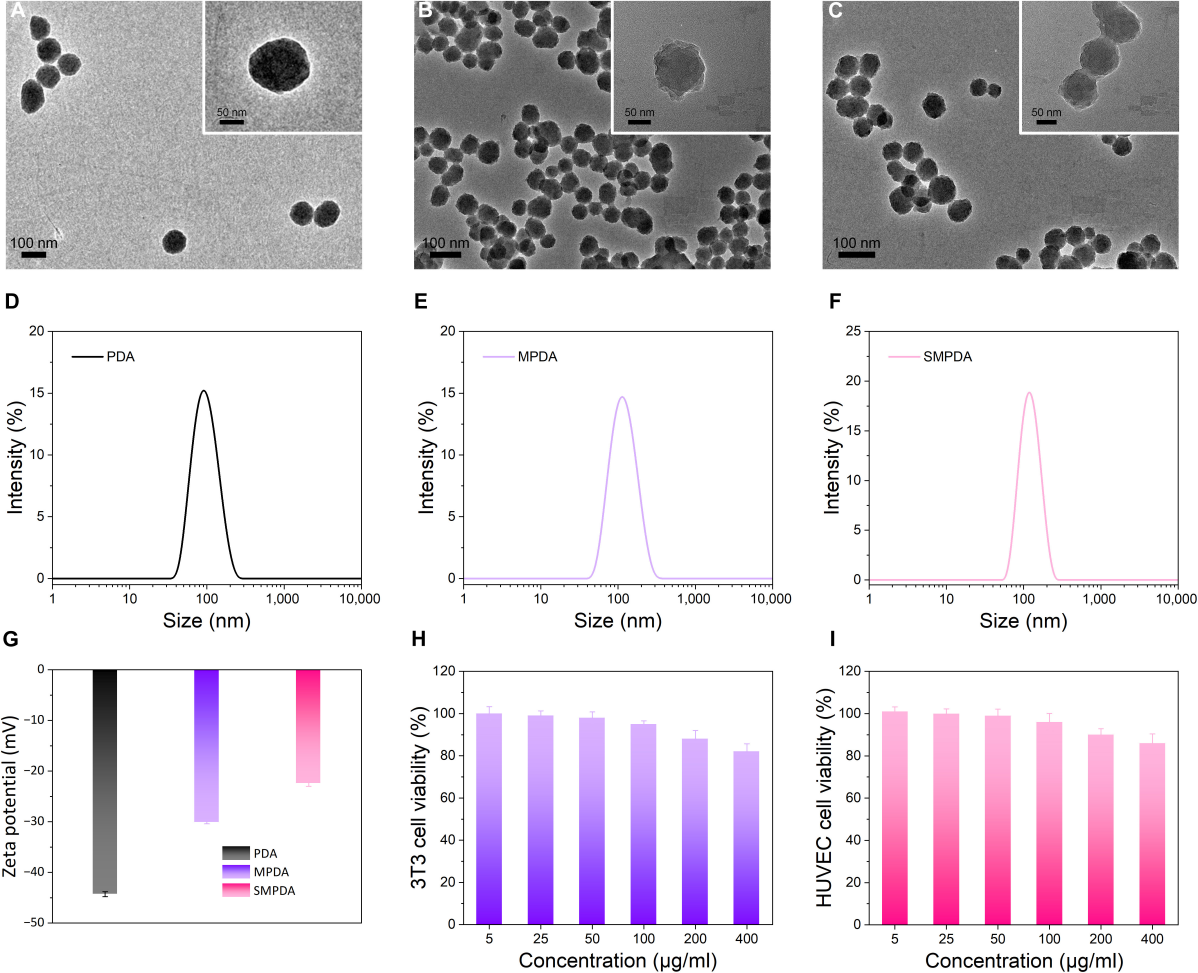
Characterization of SMPDA NPs. Transmission electron microscopy (TEM) image of (A) PDA, (B) MPDA, and (C) SMPDA. Size distribution of (D) PDA, (E) MPDA, and (F) SMPDA determined by dynamic light scattering. (G) Zeta potential of PDA, MPDA, and SMPDA (*n* = 3). (H and I) Biocompatibility evaluation of SMPDA using the CCK-8 assay (*n* = 3).

Furthermore, the examination of the morphology and particle size distribution of NPs was conducted using a transmission electron microscope and DLS analysis. TEM images of PDA, MPDA, and SMPDA are presented in Fig. 2A to C, illustrating a remarkable level of uniformity in their appearance. The DLS analysis revealed that the average diameter of MPDA and SMPDA was measured at 120 and 123 nm (Fig. 2D to F), slightly exceeding the size of PDA (110 nm). This variation in size could be attributed to the presence of mPEG on the surface of PDA. As depicted in Fig. 2G, the zeta potentials exhibited an increase from −27.2 (PDA) to −20.8 mV following the introduction of MPDA, and a similar value of −20.8 mV was observed after SNO loading on MPDA to yield SMPDA. Furthermore, the biocompatibility of SMPDA was assessed through the utilization of the CCK-8 assay. As indicated in Fig. 2H and I, the viabilities of HUVECs and mouse fibroblasts (3T3) exceeded 90% when the concentration of SMPDA did not exceed 400 μg/ml, thus highlighting the exceptional biocompatibility of the material.

### Photothermal properties of SMPDA

**Fig. 3.**
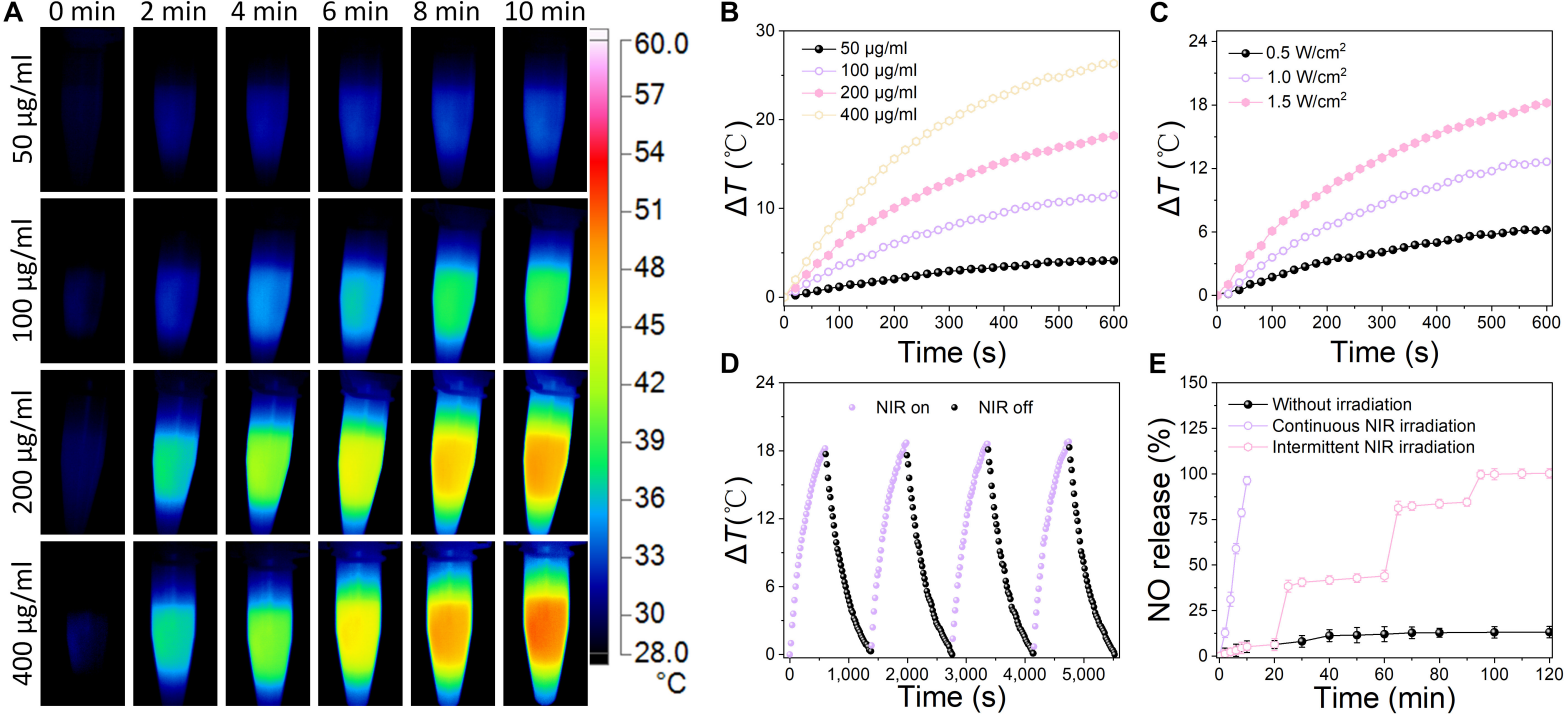
Photothermal performance of SMPDA NPs. (A) Real-time infrared thermal images and (B) photothermal curves of SMPDA solutions at various concentrations. (C) Photothermal curves of SMPDA solutions under different laser power intensities. (D) Photothermal curves of 200 μg/ml SMPDA solution in 4 on/off cycles. (E) NO release from SMPDA NPs after continuous NIR irradiation for 10 min or intermittent NIR for 5 min every 20 min, using those without NIR irradiation as control (*n* = 3).

The in vitro assessment of the photothermal properties of SMPDA involved the utilization of different NP concentrations and laser intensities. SMPDA samples were prepared at concentrations of 0, 50, 100, 200, and 400 μg/ml in PBS, followed by continuous irradiation using an 808-nm laser at an intensity of 1.5 W/cm^2^ for a duration of 10 min. Figure 3A and B illustrates that the photothermal efficiency displayed a positive correlation with both the concentration of NPs and the duration of laser irradiation. The SMPDA solution at a concentration of 400 μg/ml exhibited a substantial temperature increase, reaching up to 46.9 °C, whereas the PBS solution maintained a constant temperature of 30 °C (Fig. S4). Additionally, Fig. 3C and Fig. S5 elucidate that the temperature of SMPDA experienced a proportional increase in response to the intensity of the laser power. Specifically, the temperature reached notable values of 46.8 and 38.8 °C at laser power levels of 1 W/cm^2^ and 0.5 W/cm^2^, respectively. The photothermal characteristics of SMPDA displayed robust stability throughout 4 consecutive cycles of laser heating and cooling (Fig. 3D). This resilience further reinforces its applicability as a PTA for repetitive PTT. These observations corroborate that the control of temperature elevation can be achieved through the precise adjustment of SMPDA concentration, laser power density, and irradiation duration. Additionally, it was verified that the heat produced by SMPDA was sufficient for the effective elimination of bacteria.

The investigation into the photoactivated release of NO from SMPDA was conducted subsequent to laser irradiation, with the quantification of NO levels carried out using the Griess assay [35]. As delineated in Fig. 3E, minimal NO release was detected in the absence of laser irradiation, whereas approximately 83.6% of the loaded NO was promptly released from SMPDA NPs upon continuous exposure to an 808-nm laser for a duration of 10 min. Furthermore, the cumulative release patterns were evaluated following intermittent laser irradiation lasting 10 min at half-hour intervals, during which the temperature of the surrounding medium exhibited recurrent fluctuations. The initiation of laser irradiation at specific time points led to a sudden and substantial release of NO, which was ascribed to the thermal degradation of SNO bonds [43]. Upon discontinuation of the laser irradiation, the release of NO experienced a notable reduction, indicating the feasibility of laser-triggered NO release for applications in antibacterial contexts.

### Preparation and characterization of the SMPDA/G system

**Fig. 4.**
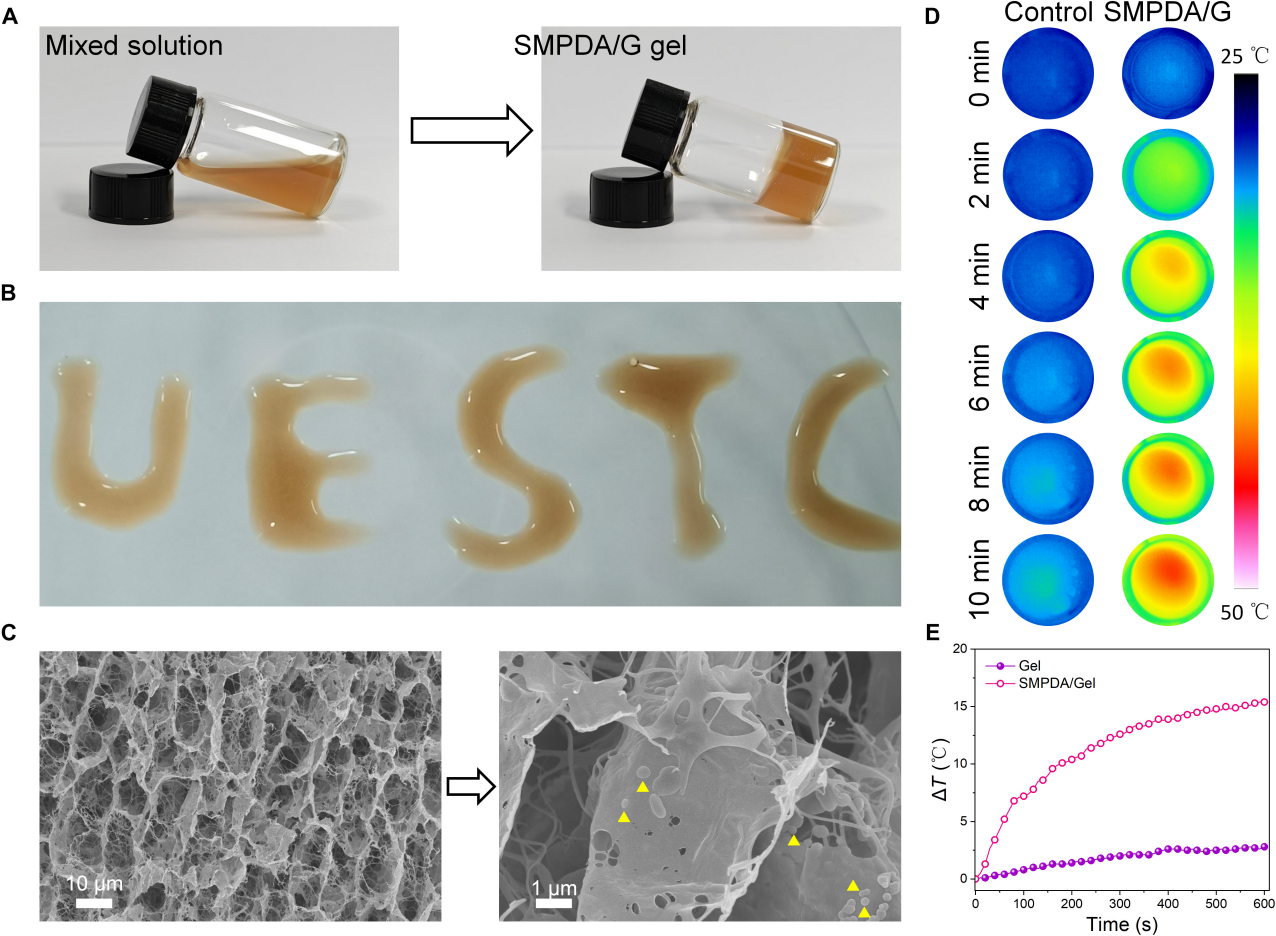
Fabrication, characterization, and photothermy of the SMPDA/G gel. (A) Optical images of the formation of the SMPDA/G hydrogel. (B) Injection of SMPDA/G into different letters. (C) SEM images of SMPDA/G hydrogels. The yellow arrowheads point to SMPDA NPs. (D) Infrared thermal images and (E) photothermal curves of the SMPDA/G irradiated with a 808-nm laser at 1 W/cm^2^ for 10 min.

The formation of FG was expedited through the concurrent extrusion and blending of equi-volume PBS solutions, one containing fibrinogen and the other containing size-optimized SMPDA NPs or thrombin. To attain the most suitable FG configuration, we initiated the process by creating a blank gel with varying ratios of fibrinogen to thrombin (specified as fibrinogen:thrombin, mg/U). Figure S6A visually presents the generation of transparent hydrogels in fibrinogen solutions with different ratios (ranging from 3:2 to 2:3) upon the combination of fibrinogen and thrombin PBS solution. The gelation times associated with these ratios are elucidated in Fig. S6B. Notably, the SEM results unveiled a porous network structure in the fibrin hydrogels, with the highest degree of uniformity in the gel structure observed when the mixing ratio was 3:2 (Fig. S6C). Hence, the ratio of 3:2 (fibrinogen:thrombin, mg/U) was deemed the optimal choice for the preparation of NP-loaded FG. Figure 4A provides an illustrative depiction of the process involved in the formation of fibrin hydrogels. The mixed solution, comprising fibrinogen containing SMPDA and thrombin, demonstrated remarkable fluidic properties, and the injectability of the SMPDA/G was validated through an injection experiment (Fig. 4B). The SEM images presented in Fig. 4C unveiled the morphology of the FG containing SMPDA NPs and displayed the uniform dispersion of NPs within the gel matrix. Afterward, the dynamic swelling ratios of the G and SMPDA/G were studied by immersing the pre-weighed freeze-dried hydrogels in PBS until reaching swelling equilibrium (Fig. S7). Equilibrium swelling ratios of G and SMPDA/G were determined to be 3.7 and 3.5, respectively, attributed to the network structure of gels. Additionally, SMPDA/G could be thoroughly degraded after 7 days at room temperature (Fig. S8). This feature ensured that when used for WH, SMPDA/G had the potential to be totally degraded and therefore did not need to be removed.

In addition, the photothermal properties of SMPDA/G were evaluated. As outlined in Fig. 4D and E, under the influence of continuous irradiation with an 808-nm laser at 1.5 W/cm^2^ for 10 min, SMPDA/G exhibited a gradual elevation in temperature, ultimately reaching 48.2 °C. This finding established the photothermal prowess of SMPDA/G, rendering it with the potential for bacterial elimination. In contrast, the control gel attained a temperature of 30.9 °C and manifested minimal photothermal effects when subjected to the same experimental conditions. Moreover, the photoactivated release of NO from SMPDA/G was examined following 808-nm laser irradiation, with the quantification of NO levels performed using the Griess assay. As illustrated in Fig. S9, minimal NO release was detected in the absence of laser irradiation. However, a substantial release of approximately 51 μM NO was promptly witnessed from SMPDA/G subsequent to continuous 808-nm laser irradiation for a duration of 10 min, suggesting laser-triggered NO release for antibacterial applications.

### In vitro antibacterial performance and immunomodulating properties

BIs represent a primary contributor to the persistence of non-healing wounds. Consequently, the antimicrobial properties assume paramount significance in wound dressings. In this regard, we initiated an investigation to confirm the in vitro antibacterial effectiveness and immunomodulatory features of these biomaterials. Specifically, the antibacterial potential of SMPDA against *Escherichia coli* (gram-negative) and *S. aureus* (gram-positive) was explored. As delineated in Fig. S10A to D, no pronounced bac-terial toxicity was witnessed across all treatment groups in the absence of irradiation. However, compared to the control groups (PBS+NIR), the number of *E. coli* and *S. aureus* could be killed by SMPDA after NIR irradiation, and the number of colonies gradually decreased as the concentration of SMPDA increased, suggesting that SMPDA killed bacteria in a dose-dependent manner.

**Fig. 5.**
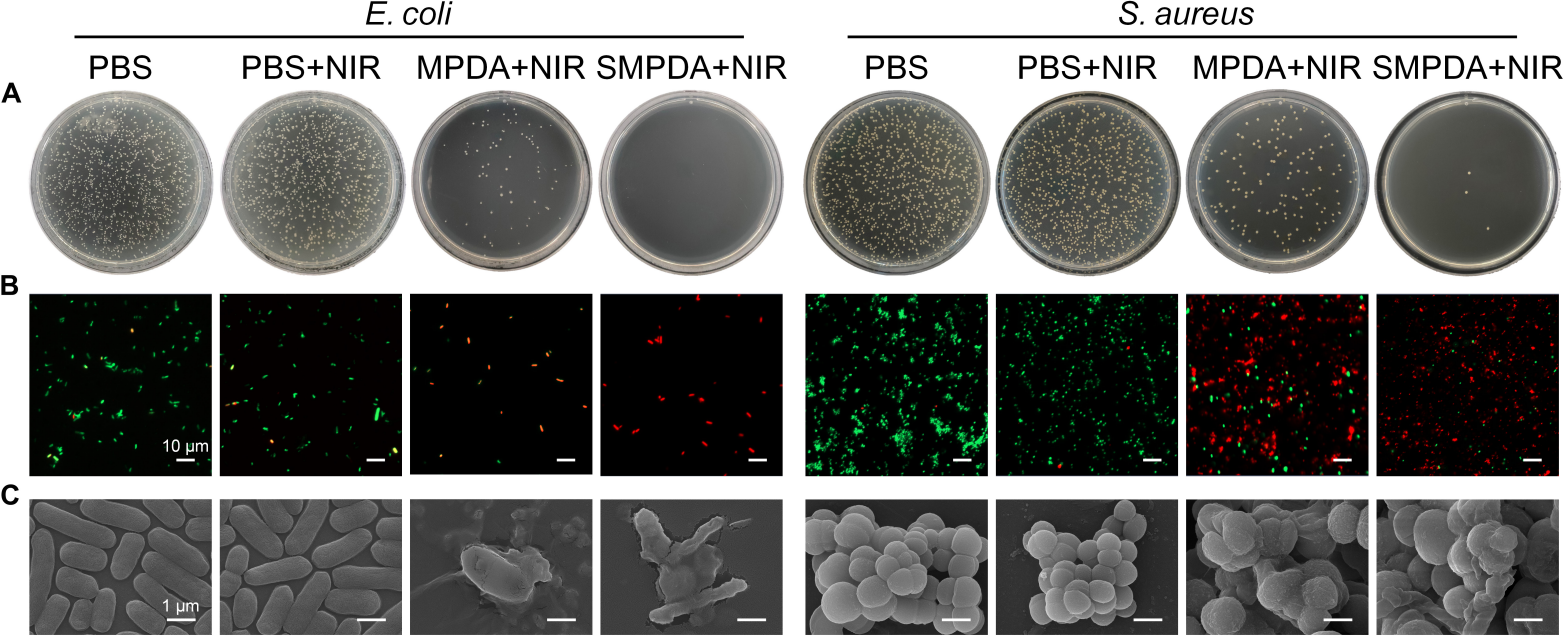
In vitro antibacterial properties of NPs. (A) Bacterial colonies, (B) live/dead staining, and (C) SEM imaging of *E. coli* and *S. aureus* after different treatments.

In addition, upon subjecting the MPDA, SMPDA, and PBS groups to irradiation with an 808-nm laser operating at 1.5 W/cm^2^ for 5 min, SMPDA showcased an exceptional capacity for antibacterial activity, with the complete absence of bacterial colony formation (Fig. 5A). This observation underscores the synergistic interplay between the NO and PTT to SMPDA. The antibacterial potential was subjected to additional examination through the implementation of a live/dead bacterial staining assay. PI dye was utilized to selectively label bacteria with disrupted structural integrity, resulting in a red fluorescence upon excitation. Conversely, the SYTO 9 dye was employed to target live bacteria by binding to their DNA, resulting in a green fluorescence when excited. As depicted in Fig. 5B, the SMPDA+L group displayed a prominently higher percentage of red fluorescence in comparison to the control group. This difference served as robust validation that SMPDA, when subjected to 808-nm laser irradiation, could effectively eradicate *E. coli* and *S. aureus*. In addition, to gain deeper insights into the bactericidal potential of SMPDA, we observed the morphological alterations undergone by bacteria upon exposure to SMPDA under 808-nm laser irradiation. The experimental results delineated in Fig. 5C provide additional affirmation that SMPDA with 808-nm laser irradiation effectively disrupts bacterial structures through a synergistic interplay involving NO and PTT for bacterial eradication.

Bacterial biofilm is characterized by an organized bacterial community enclosed within a self-generated extracellular polymeric matrix [44]. The formation of biofilms is a pivotal factor contributing to enduring BIs, which exert a substantial hindrance to WH. Accordingly, we undertook an assessment of the antimicrobial potential of SMPDA against biofilms. As displayed in Fig. S11, the development of biofilms was markedly curbed in the SMPDA+L group. These findings establish that SMPDA exhibits exceptional antimicrobial efficacy against both planktonic bacteria and biofilms.

**Fig. 6.**
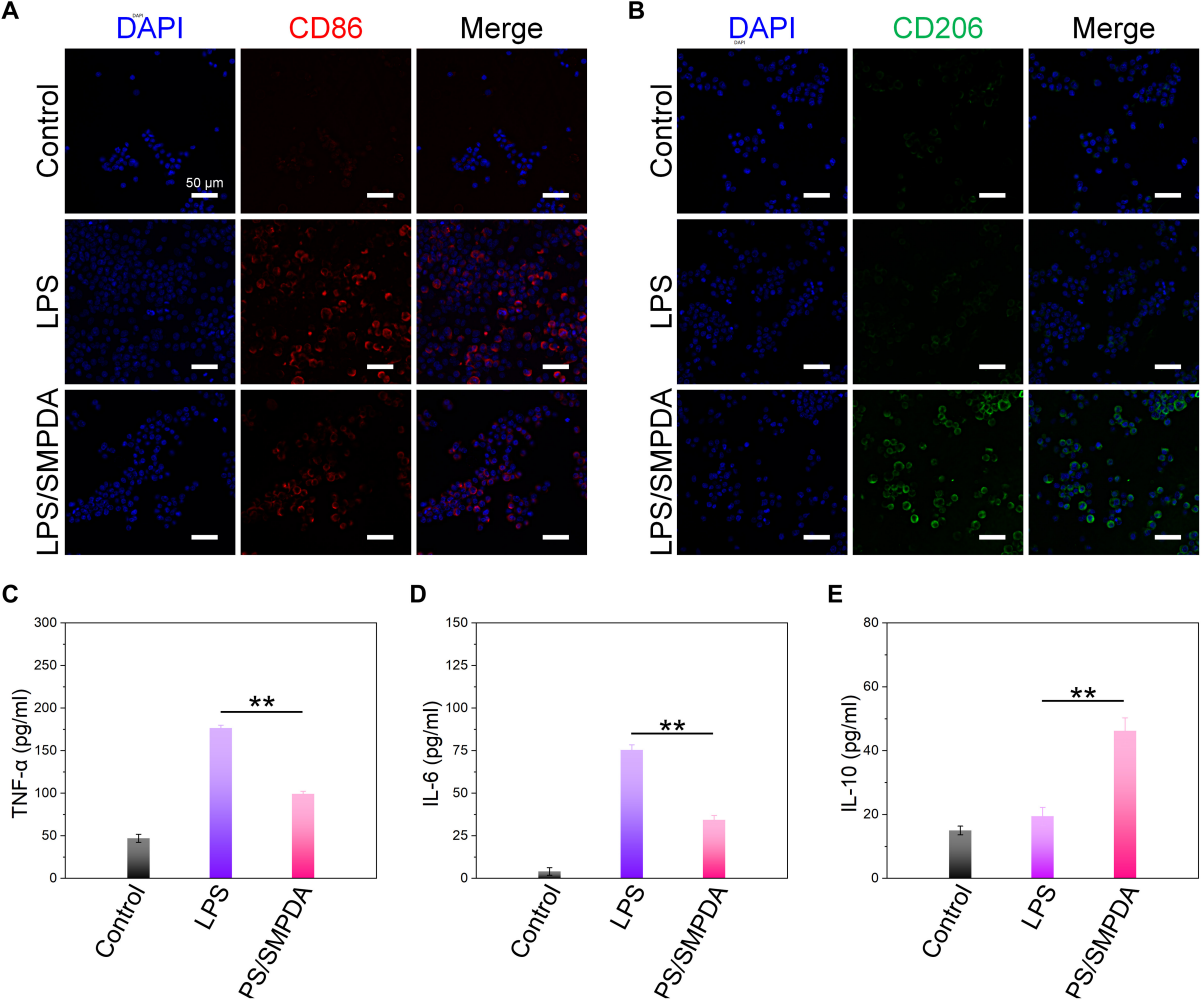
In vitro immunomodulating activity evaluation. (A and B) Fluorescence images of RAW 264.7 cells after treatment with PBS (control), LPS, or LPS+SMPDA (*n* = 3). Statistical analysis of (C) TNF-𝛼, (D) IL-6, and (E) IL-10 ELISA data (*n* = 3) (**P < 0.01).

The dysregulation of the immune response is a contributing factor to impaired WH, and chronic wounds are often characterized by a sustained pro-inflammatory state [45]. Effective modulation of the IME holds promise as a treatment strategy for chronic wounds [33]. In the wound site, M1 macrophages are responsible for producing pro-inflammatory cytokines, while M2 macrophages exhibit an anti-inflammatory phenotype and release anti-inflammatory mediators. PDA, characterized by its exceptional biocompatibility, can hinder M1 polarization while facilitating M2 polarization [31]. Subsequently, we proceeded to assess the immunomodulatory characteristics of SMPDA. Surface markers CD86 and CD206 are expressed by M1 and M2 macrophages, respectively. Notably, CD206 expression displayed a substantial increase in the SMPDA group, while CD86 expression exhibited a significant decrease in the same group (Fig. 6A and B). Among the key anti-inflammatory cytokines produced by M2 macrophages, interleukin (IL)-10 held particular significance. Conversely, the pro-inflammatory cytokines IL-6 and tumor necrosis factor-𝛼 (TNF-𝛼) were predominantly produced by M1 macrophages. The findings revealed pronounced suppression in IL-6 and TNF-𝛼 levels in the SMPDA group (Fig. 6C and D) compared to the control group. Conversely, IL-10 displayed a marked elevation in the SMPDA group (Fig. 6E). These findings imply that SMPDA fosters the polarization of M2 macrophages while restraining the polarization of M1 macrophages, consequently promoting an anti-inflammatory response.

### SMPDA/G accelerates the infected WH through sterilization in vivo

**Fig. 7.**
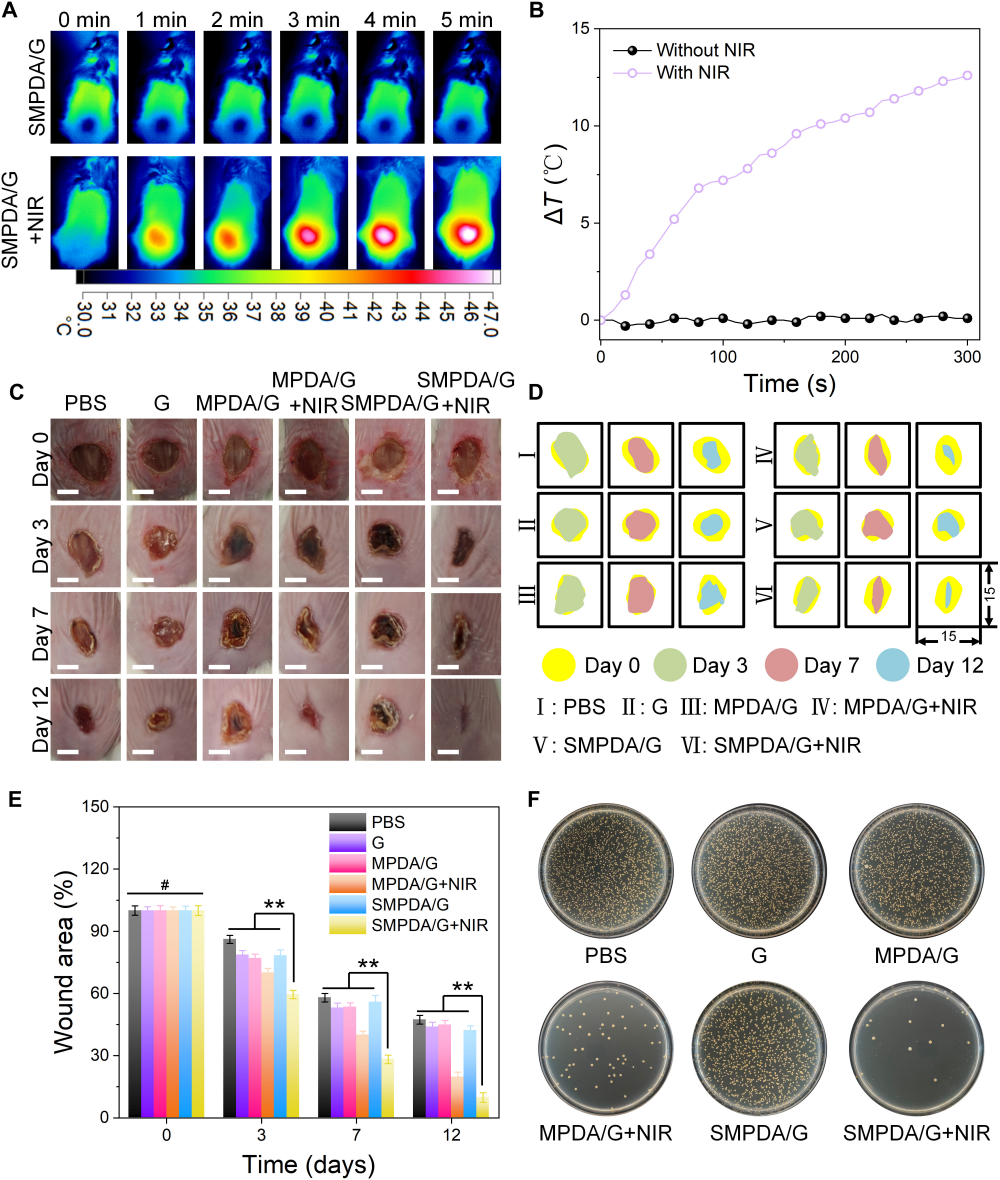
Therapeutic effects of SMPDA/G gel in promoting infected wound healing. (A) Thermal imaging images of mice under different treatments (808 nm, 1.5 W/cm^2^, 5 min). (B) Corresponding changes in wound maximum temperature. (C) Optical images of wounds in different treatment groups on days 0, 3, 7, and 12. The scale bar is 1 cm. (D) Changes in wound area from day 0 to day 12. (E) Wound healing rate data statistics in the PBS, G, MPDA/G,MPDA/G+NIR, SMPDA/G, and SMPDA/G+NIR groups (^#^*P* > 0.05, **P < 0.01). (F) Bacterial colonies for trauma on day 6.

The WH effectiveness of SMPDA/G was additionally confirmed through in vivo models of bacteria-infected wounds. Our choice of the *S. aureus*-infected wound model for further verification was based on the prevalence of *S. aureus* as a causative agent in wound infections. The molding and gel injection process is visually depicted in Fig. S12. Our initial assessment in the in vivo context focused on the thermal effects of SMPDA/G. As delineated in Fig. 7A and B, upon exposure to an 808-nm laser at 1.5 W/cm^2^ for 5 min, SMPDA/G experienced a controlled temperature elevation to 46 °C. This mild photothermal effect was conducive to bacterial eradication while preserving the integrity of the wound tissue [46]. Subsequently, the status of the wounds was documented on days 0, 3, 7, 10, and 12. The wound closure rates were prominently higher in the MPDA+L and SMPDA/G+L groups compared to the other experimental groups (Fig. 7C and D). Quantitative analysis unveiled noteworthy reductions in relative wound areas, reaching 47.35%, 43.88%, 44.83%, 19.65%, 42.11%, and 9.82% on day 12 for the PBS, G, MPDA/G, MPDA/G+L, SMPDA/G, and SMPDA/G+L groups, respectively (Fig. 7E). Importantly, no pronounced differences in the weight change of the mice were witnessed across all treatment groups during the entire treatment duration (Fig. S13). These findings collectively highlight the enhanced potential of the MPDA/G+L and SMPDA/G+L groups in fostering WH under infected conditions. Furthermore, due to the synergistic interplay between NO and PTT, it became evident that the SMPDA/G+L group outperformed the MPDA/G+L group in terms of its WH capabilities. To provide further validation of the antibacterial effect of SMPDA/G in vivo, wound fluid was collected and subjected to bacterial quantification through a colony formation assay on day 6. A marked reduction in the number of bacterial colonies was evident in the MPDA/G+L and SMPDA/G+L groups, with the SMPDA/G+L group exhibiting a scarcity of bacterial colonies on day 6 post-treatment (Fig. 7F). These findings underscore the synergistic antibacterial effects of SMPDA/G, attributed to the combined actions of NO and PTT, thereby accelerating the process of WH.

**Fig. 8.**
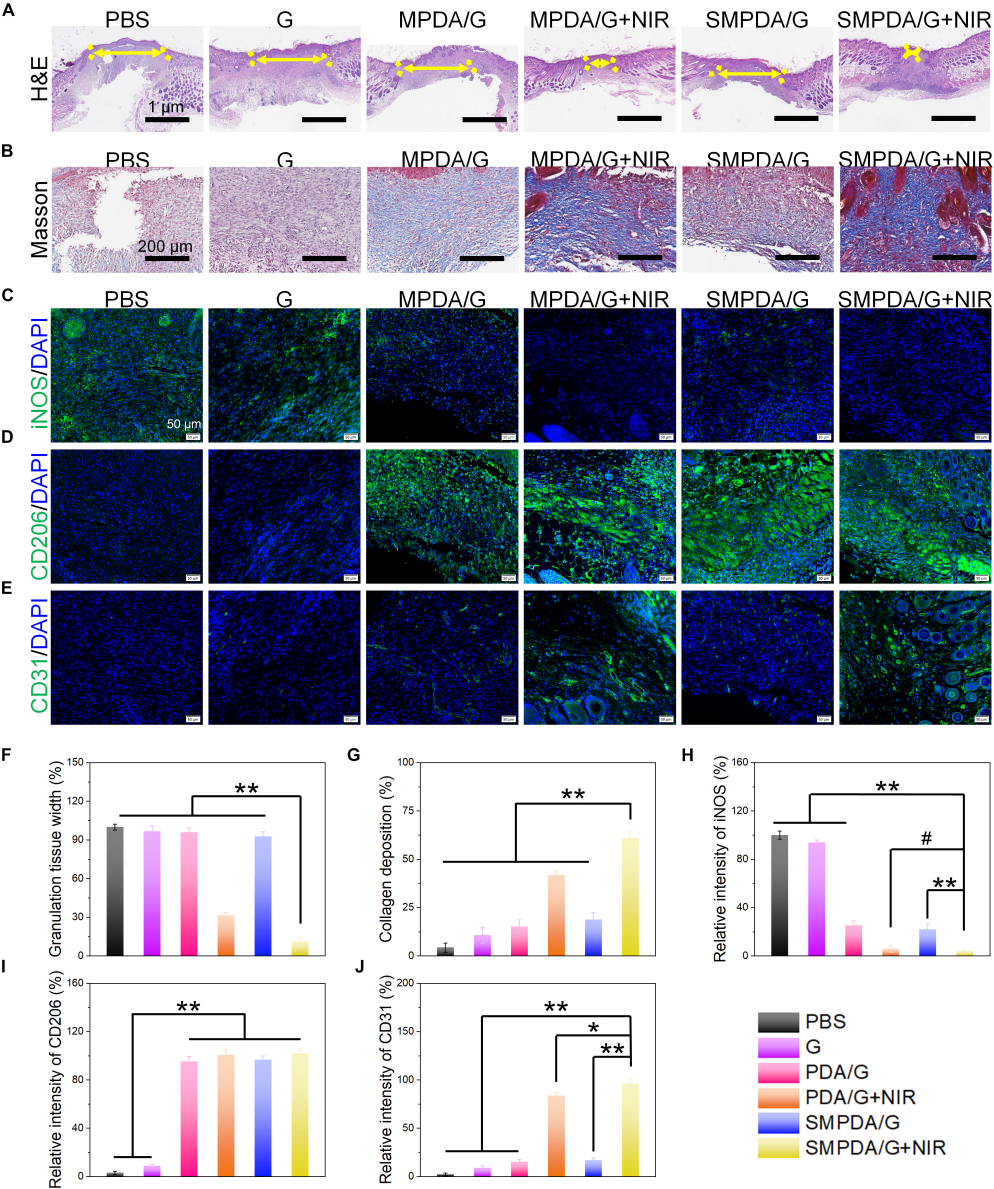
Analysis of tissue section staining data. (A) Representative images of HE staining of the wound bed. The double-headed arrows indicate the granulation tissue gaps and wound edges. (B) Masson staining of collagen deposition. Immunofluorescence staining of (C) iNOS, (D) CD206, and (E) CD31 in skin tissue with different treatments. (F to J) Quantitative analysis of stained images acquired from different groups: scar width, college deposition, iNOS, CD206, and CD31 (*n* = 3, ^#^*P* > 0.05, *P < 0.05, and **P < 0.01), respectively.

The assessment of WH post-treatment was carried out through histological examination of the wound bed tissues utilizing hematoxylin and eosin (H&E) staining. The presence and characteristics of tissue granulation and re-epithelialization are considered pivotal indicators of the regenerative phase in the WH process. Granulation tissue, positioned between the wound margins, serves as a significant metric, wherein a narrower intergranulation tissue gap is indicative of expeditious wound closure and enhanced WH dynamics [47]. Furthermore, the migration of epithelial cells from the wound confers a pivotal role in instigating the process of re-epithelialization [48]. A substantial reduction in the inter-granulation tissue gap in the SMPDA+L group was noted, indicative of an expedited WH process. Remarkably, complete re-epithelialization of the wound bed was achieved in the SMPDA/G+L group (Fig. 8A and B). Additionally, the deposition of collagen exhibited a denser and more organized pattern in the SMPDA/G+L group (Fig. 8C and D). Collectively, these findings indicate that SMPDA/G+L treatment not only effectively eradicates bacteria but also stimulates prompt wound closure and enhanced collagen deposition, thereby prominently ameliorating infected WH.

### In vivo immunomodulatory property of SMPDA/G

The facilitation of macrophage transition from a pro-inflammatory to an anti-inflammatory phenotype, with subsequent modulation of the IME, represents a promising therapeutic strategy for addressing chronic wounds [49]. The assessment of wound inflammation on day 12 post-treatment involved an exploration into the inflammatory status, including macrophage polarization and inflammatory cytokine production in the wound bed. This analysis was achieved through immunofluorescence staining targeting iNOS, CD206, and 4′,6-diamino-2-phenylindole. As demonstrated in Fig. 8E and F, the NP-loaded FG groups exhibited diminished iNOS expression and heightened CD206 expression, signifying that the utilization of MPDA NPs contributed to an enhanced polarization of M2 macrophages and a reduction in inflammation. Considering the vital role of neovascularization in furnishing ample oxygen and nutrients crucial for tissue repair, it assumes a pivotal function in WH and tissue regeneration throughout the proliferative phase. Thus, the neovascularization in the granulation tissue was examined through platelet endothelial cell adhesion molecule-1 (CD31) immunostaining. Relative to the PBS, G, MPDA/G, and SMPDA/G groups, the MPDA/G+L and SMPDA/G+L groups demonstrated escalated CD31 expression in the wound surface.

### In vivo biosafety property of SMPDA/G

The study included histological analyses to ascertain whether the gel induced tissue damage, inflammation, or lesions. As depicted in Fig. S14, no pronounced differences were noted between the groups subjected to gel treatment and the control group. In addition, blood biochemical tests were carried out to validate the outcomes of the histological examination and to quantitatively examine the impact of gel on the mice under study. The comparison between the control group and the SMPDA/G+L treatment group did not reveal any significant differences (Fig. S15). This observation, in conjunction with the results obtained from H&E staining and blood biochemical tests, supports the conclusion that SMPDA/G exhibits minimal toxicity and can be considered safe for potential clinical use.

Moreover, the assessment of hemolysis ratios was conducted for various gels, including G, MPDA/G, and SMPDA/G, with PBS serving as the negative control group and toxin as the positive control group. The findings, as depicted in Fig. S16, indicated that the untreated free toxin displayed a heightened hemolytic capability, which notably decreased when the toxin was replaced by the gel. These findings provide confirmation of the satisfactory blood compatibility exhibited by the FGs.

## Conclusion

In conclusion, this study presented a novel antibacterial platform referred to as SMPDA/G. Initially, SMPDA NPs were constructed through the surface deposition of a NO donor known as SNO onto MPDA NPs. Thereafter, these SMPDA NPs were uniformly incorporated into a fibrin hydrogel matrix using a double-barreled syringe. Our work establishes that SMPDA/G displays the capacity for NO release and PTT upon exposure to 808-nm laser irradiation, resulting in a synergistic antimicrobial impact. Furthermore, MPDA demonstrates its potential as an immunomodulator to foster the polarization of M2-phenotype macrophages and the secretion of anti-inflammatory factors, thus offering potential benefits for WH. Taken together, these characteristics highlight SMPDA/G as a highly promising candidate for clinical use in addressing infected wounds.

## Ethics Approval

The animal experiments were approved by the Experimental Animal Ethics Committee of West China Hospital of Sichuan University (IACUC Number: 20231103009).

## Data Availability

All data needed to evaluate the conclusions of the study are present in the paper and Supplementary Materials.
