# Supplementary material for: Injectable and Near-Infrared Light-Controllable Fibrin Hydrogels with Antimicrobial and Immunomodulating Properties for Infected Wound Healing
Source: Biomater Res. 2024 Jun 27;28:0019. doi: 10.34133/bmr.0019 (PMC11210386; doi:10.34133/bmr.0019)
Supplement: Supplementary 1 — Figs. S1 to S16 [file bmr.0019.f1.zip › Supplementary Material.docx]

Supporting Information

**Injectable and near-infrared light-controllable fibrin hydrogels with antimicrobial and immunomodulating properties for infected wound healing**

Qing Zhang^a,b,#^, Yongxian Jiang^c,#^, Xiaolong Zhang^b,#^, Yi Wang^a^, Rong Ju^b^ and Guoqing Wei^b,^*

^a^School of Life Science and Engineering, Southwest Jiaotong University, Chengdu 610031, China;

^b^Chengdu Women’s and Children’s Central Hospital, School of Medicine, University of Electronic Science and Technology of China, Chengdu 611731, China;

^c^Sichuan Provincial Matenity and Child Health Care Hospital, the Affiliated Women's and Children's Hospital of Chengdu Medical College, Chengdu 610041, China.

^#^These authors contributed equally to this work.

*Corresponding authors. E-mail addresses: [guoqing@uestc.edu.cn](mailto:guoqing@uestc.edu.cn) (G. Wei).

**
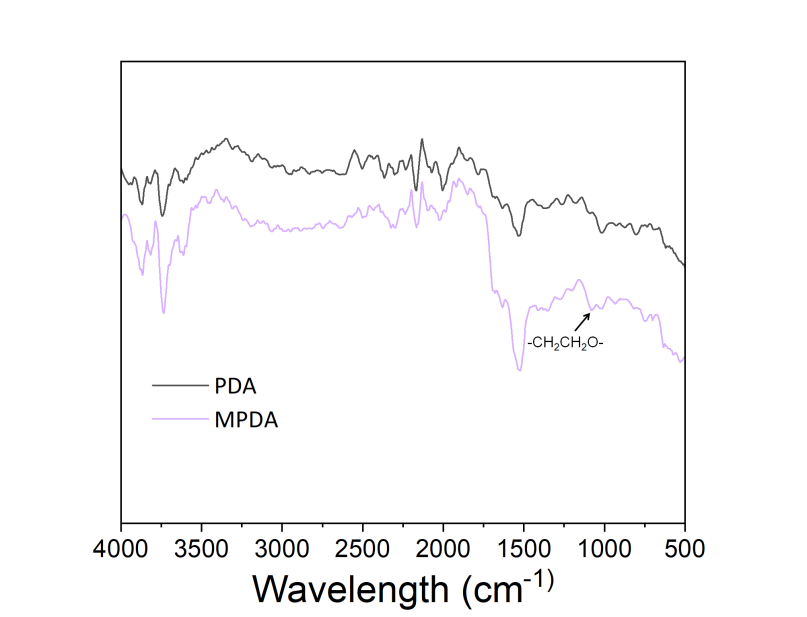
**

**Fig. S1.** FT-IR spectra analyses of PDA before and after modification mPEG on their surface.


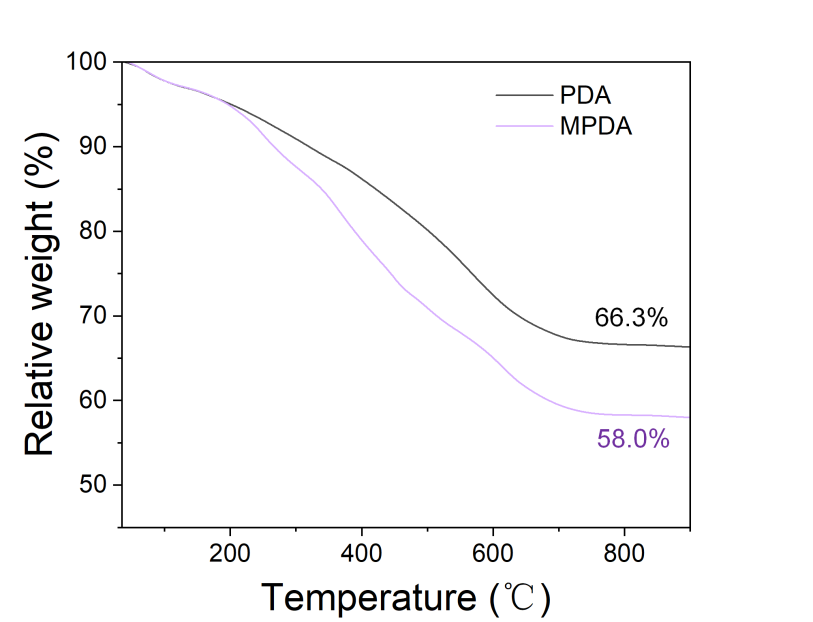


**Figure S2.** Thermal gravimetric analyses of PDA before and after modification mPEG on their surface.


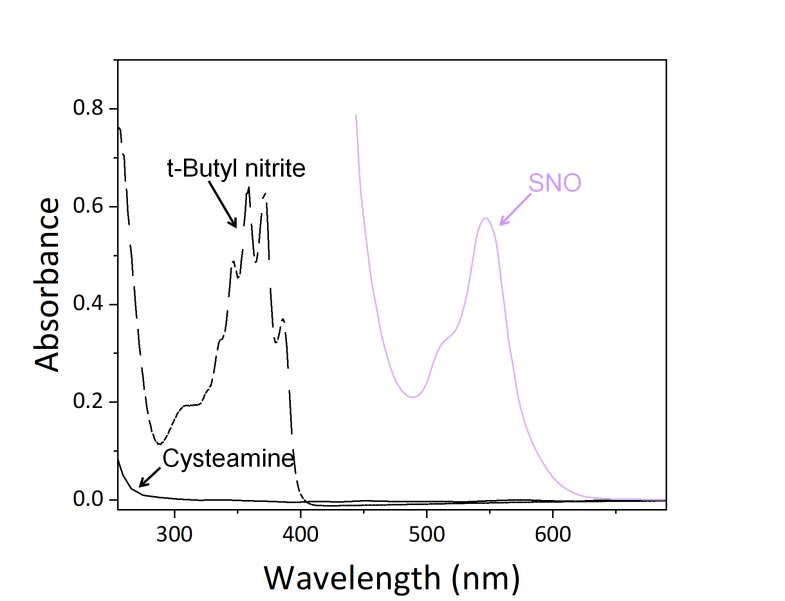


**Figure S3.** UV–vis absorption spectra of cysteamine, t-butyl nitrite, and SNO.


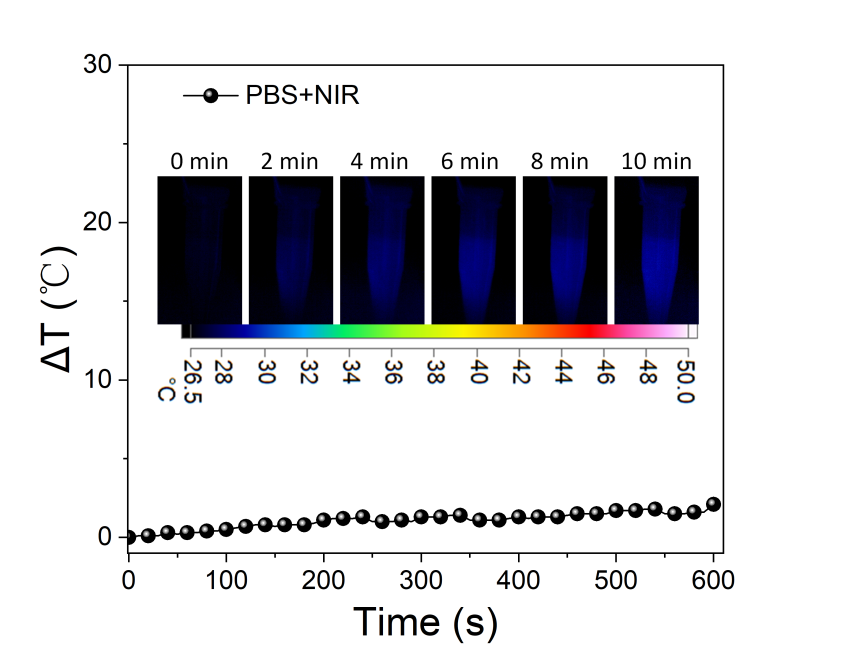


**Figure S4.** Real-time infrared thermal images and photothermal curves of PBS solutions irradiated with a 808 nm laser at 1.5 W/cm^2^.


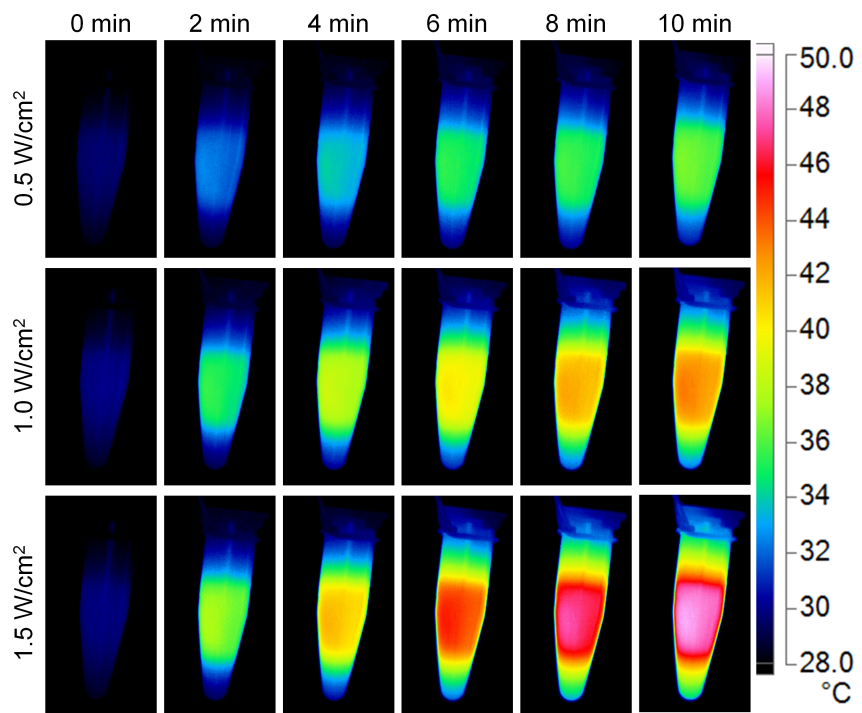


**Fig. S5** Real-time infrared thermal images SMPDA solutions under different laser power intensity.


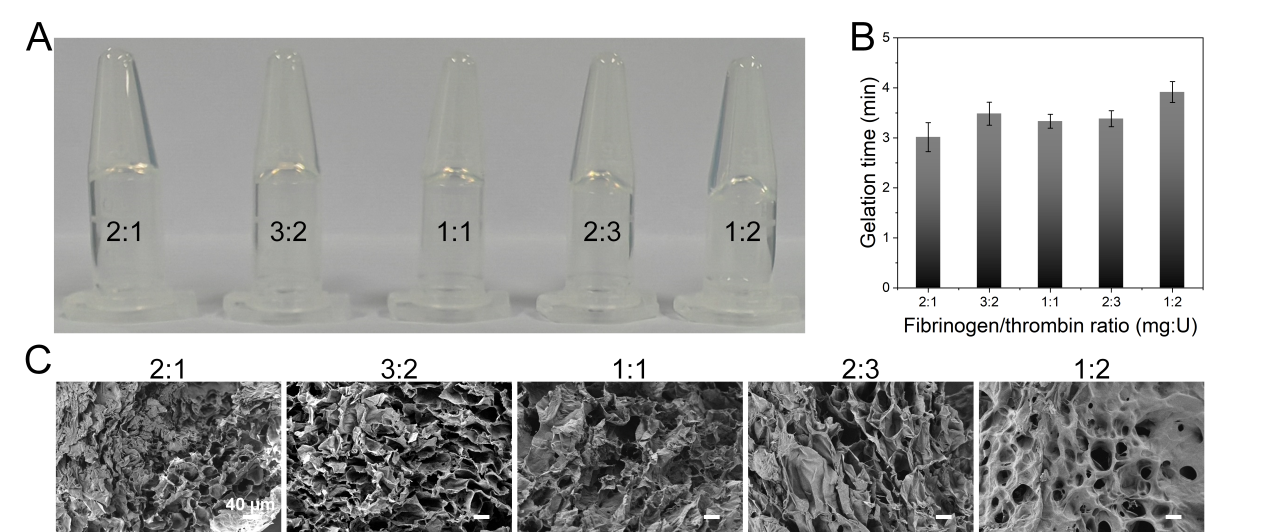


**Fig. S6** (A) Digital photos, (B) The gelation times (n = 3) and (C) SEM images of different proportions fibrin gels.


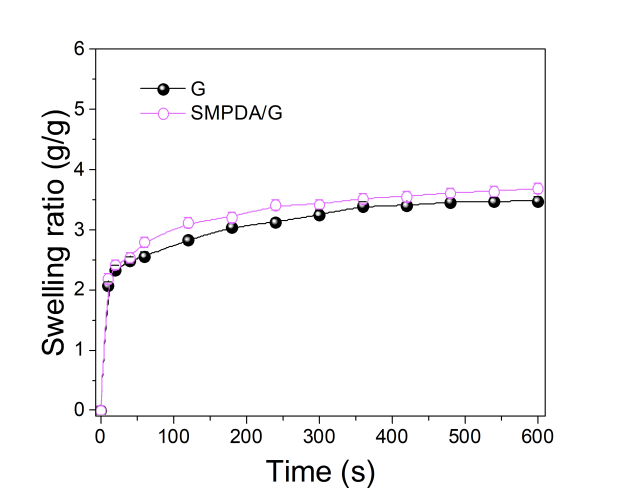


**Fig. S7** Swelling ratios of G and SMPDA/G gels (n = 3).


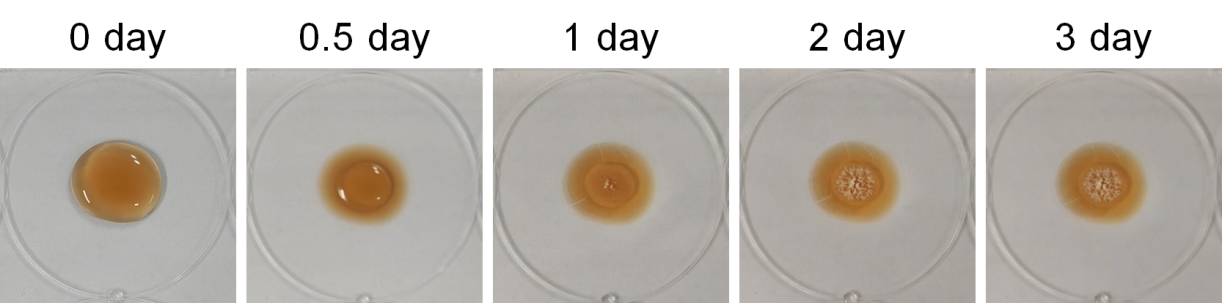


**Fig. S8** Optical images of the in vitro degradation process of SMPDA/G gel.


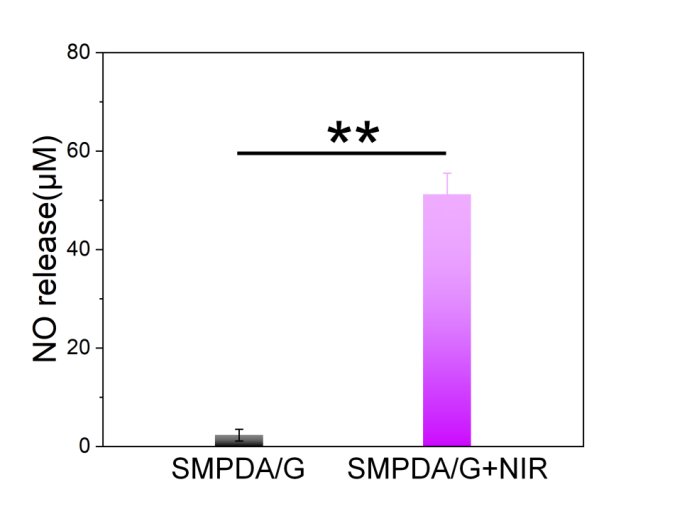


**Fig. S9** In vitro NIR controlled release curve of NO from SMPDA/G gel.


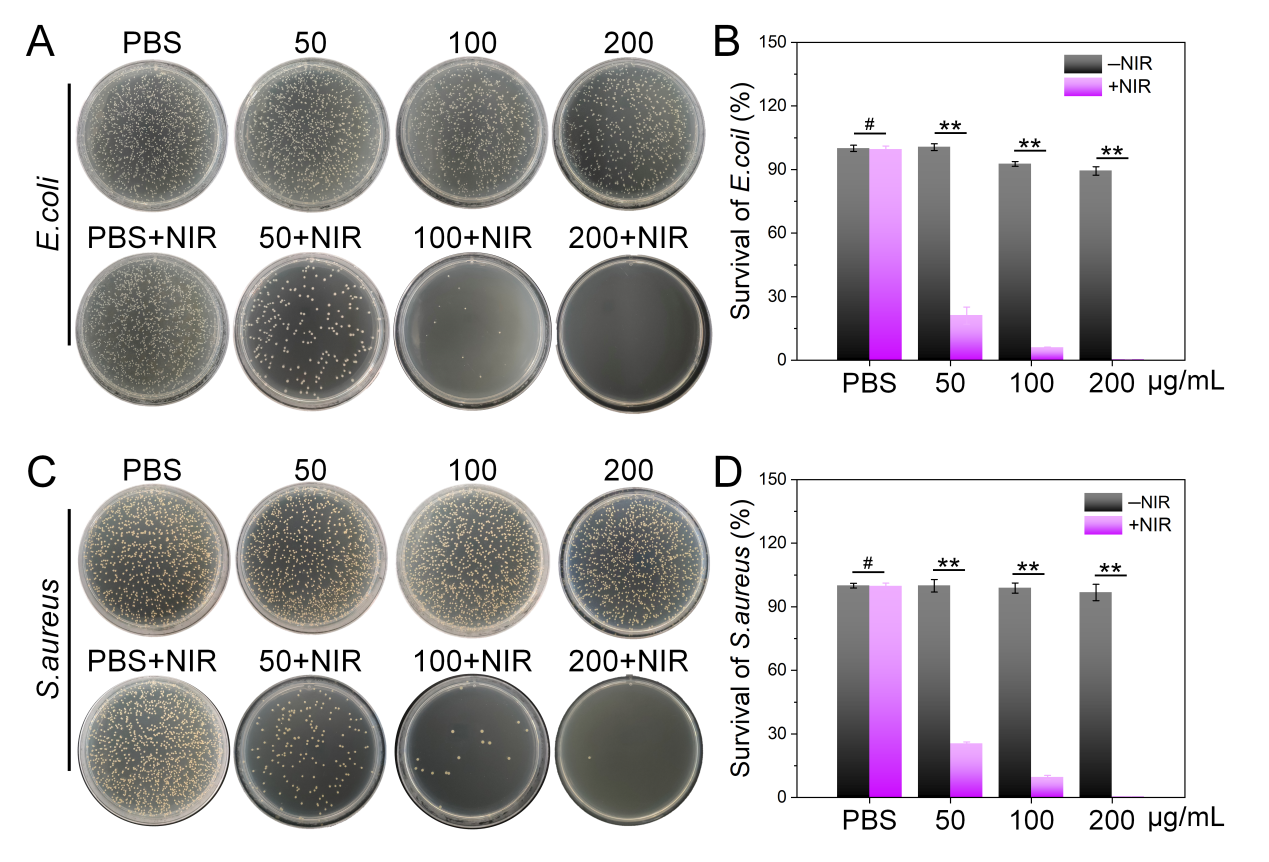


**Fig. S10** In vitro antibacterial properties of SMPDA. (**A**) and (**B**) Bacterial colonies, (**C**) and (**D**) Bacterial survival of *E. coli* and *S.aureus* after different treatments (n = 3).


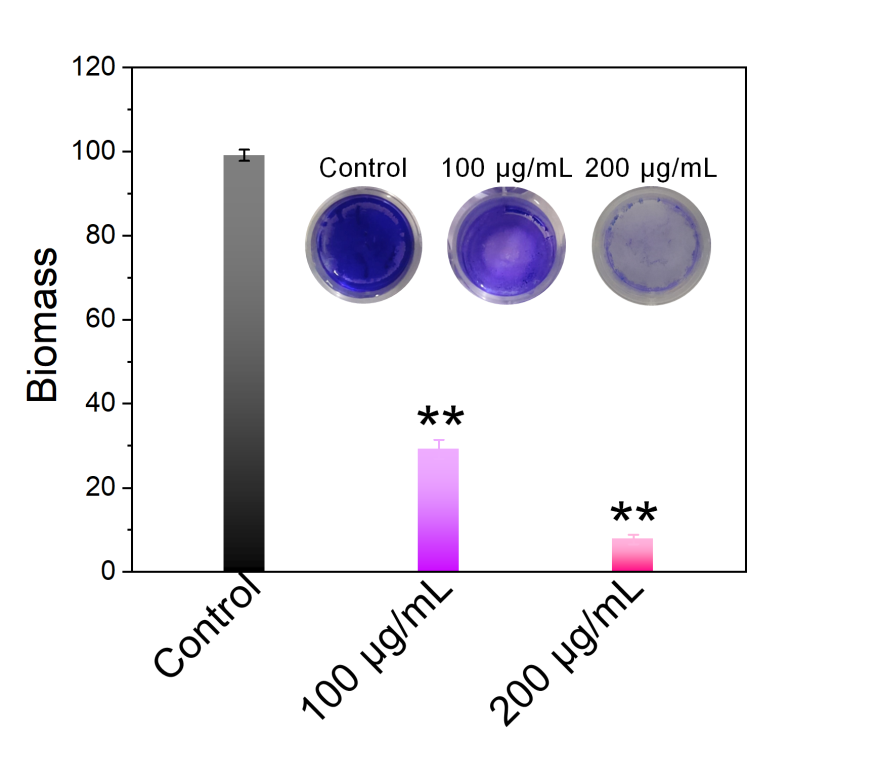


**Fig. S11** Statistical analysis of bacterial biofilm removal by SMPDA (n = 3).


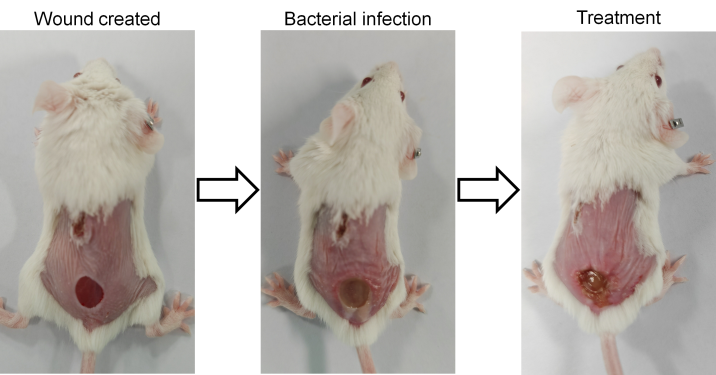


**Fig. S12** Optical images after constructing the infected mice model and SMPDA/G gel treatment.

**
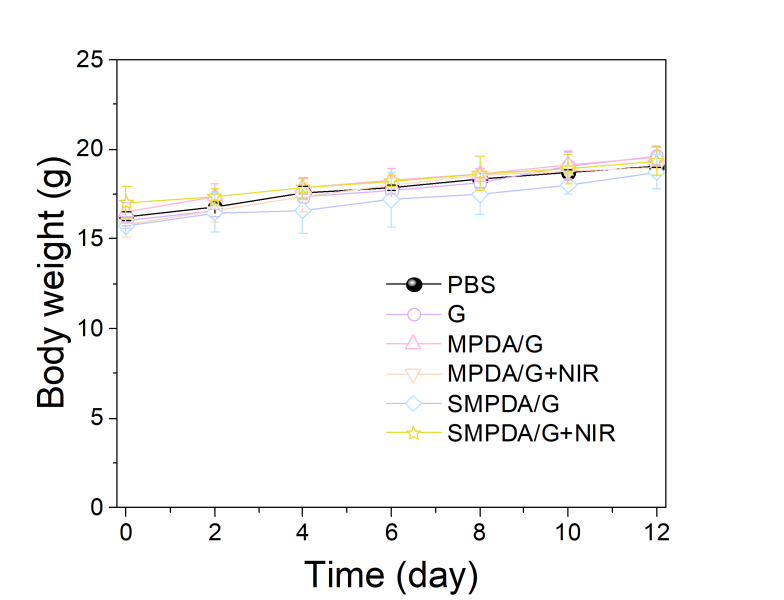
**

**Fig. S13** Curves of body weight of mice in each treatment group (n=5).

**
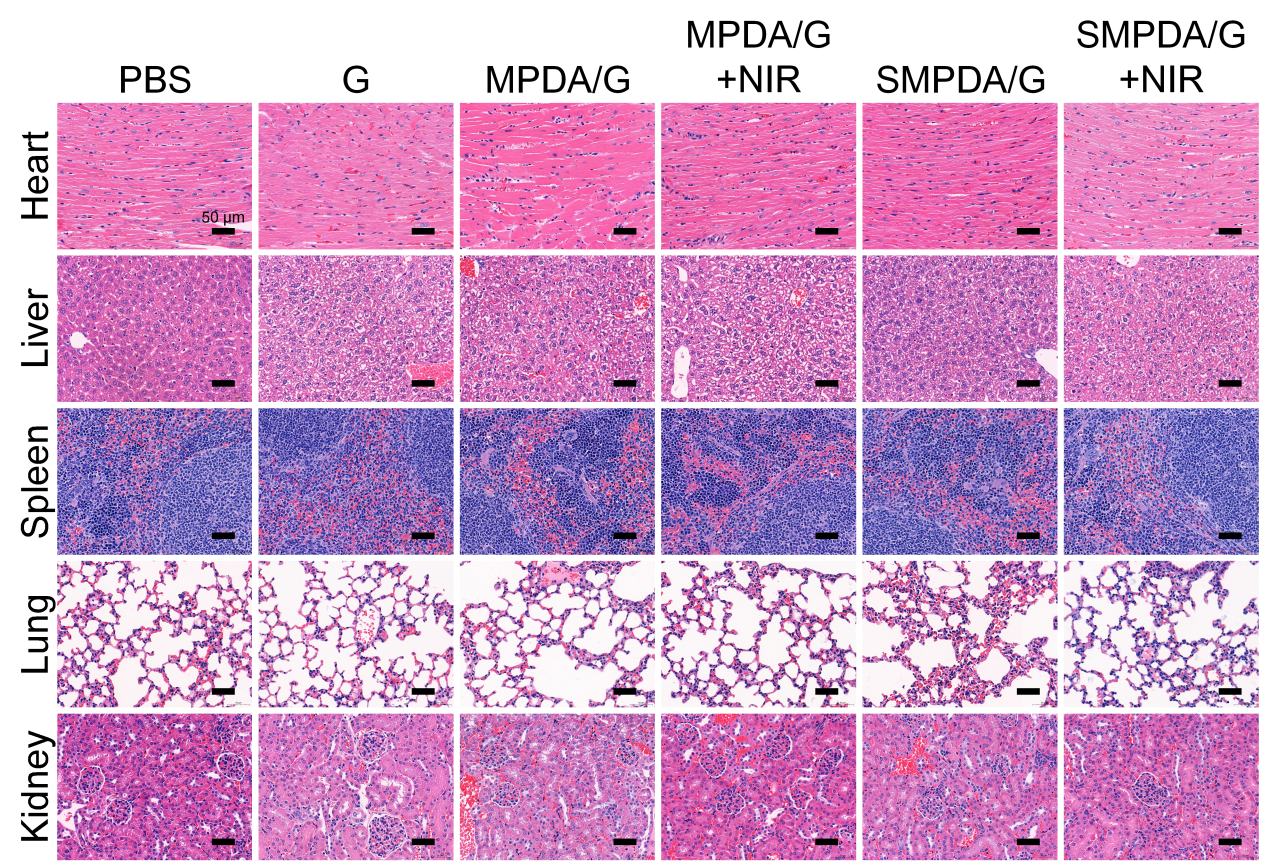
**

**Fig. S14** H&E staining of major organ tissues of experimental mice in the different treatment groups.

**
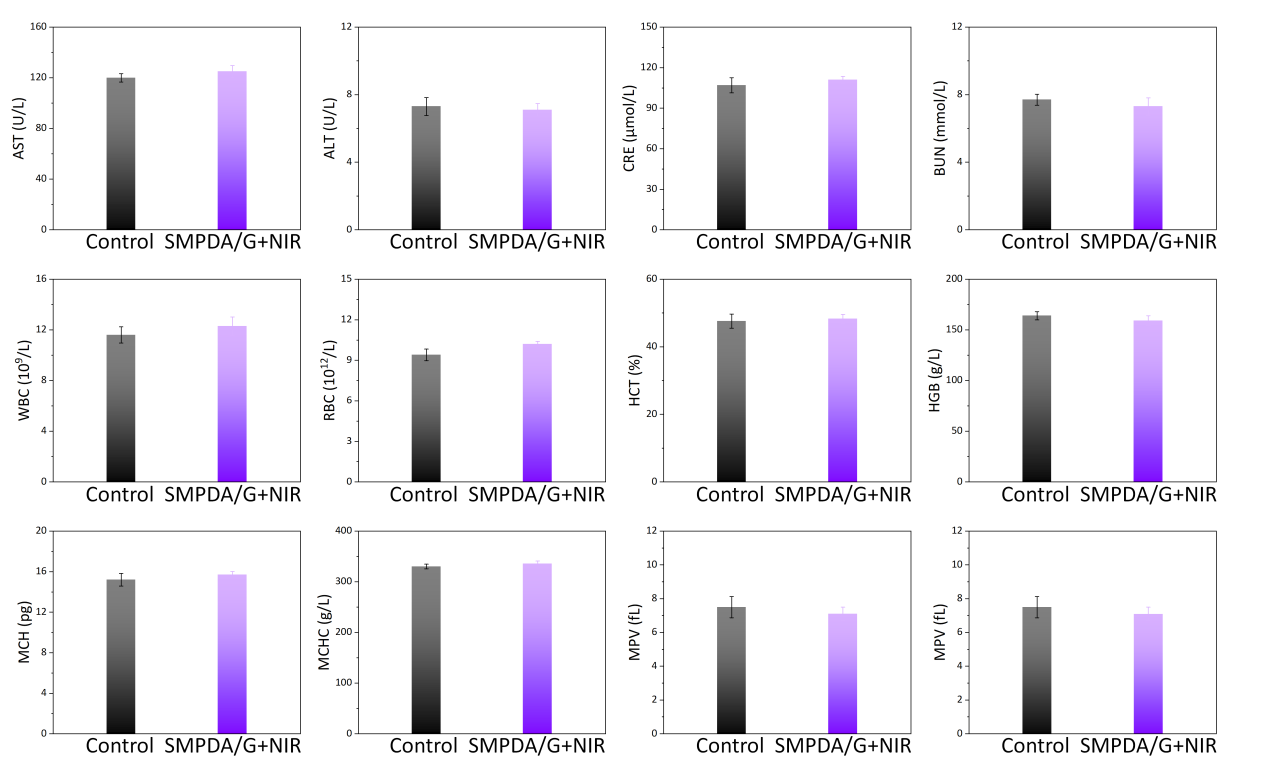
**

**Fig. S15** Blood routine and Biochemical levels after different treatments (n=3).

**
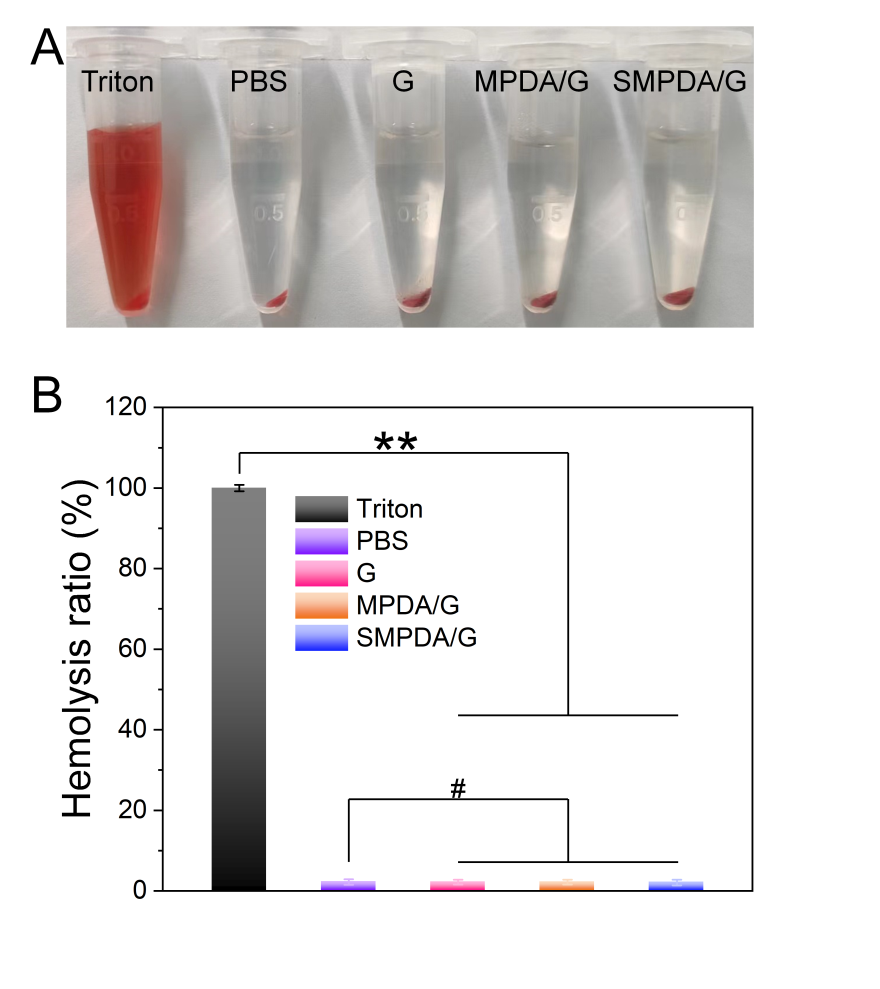
**

**Fig. S16** (**A**) Photographs from hemolysis test on Triton, PBS, G, MPDA/G, and SMPDA/G (n = 3). (**B**) Hemolysis ratios of G, MPDA/G, and SMPDA/G hydrogels (n = 3) (^#^, *P* > 0.05 and **, *P* < 0.01).
