# Supplementary figures and images for: Injectable and Near-Infrared Light-Controllable Fibrin Hydrogels with Antimicrobial and Immunomodulating Properties for Infected Wound Healing
Source: Biomater Res. 2024 Jun 27;28:0019. doi: 10.34133/bmr.0019 (PMC11210386; doi:10.34133/bmr.0019)

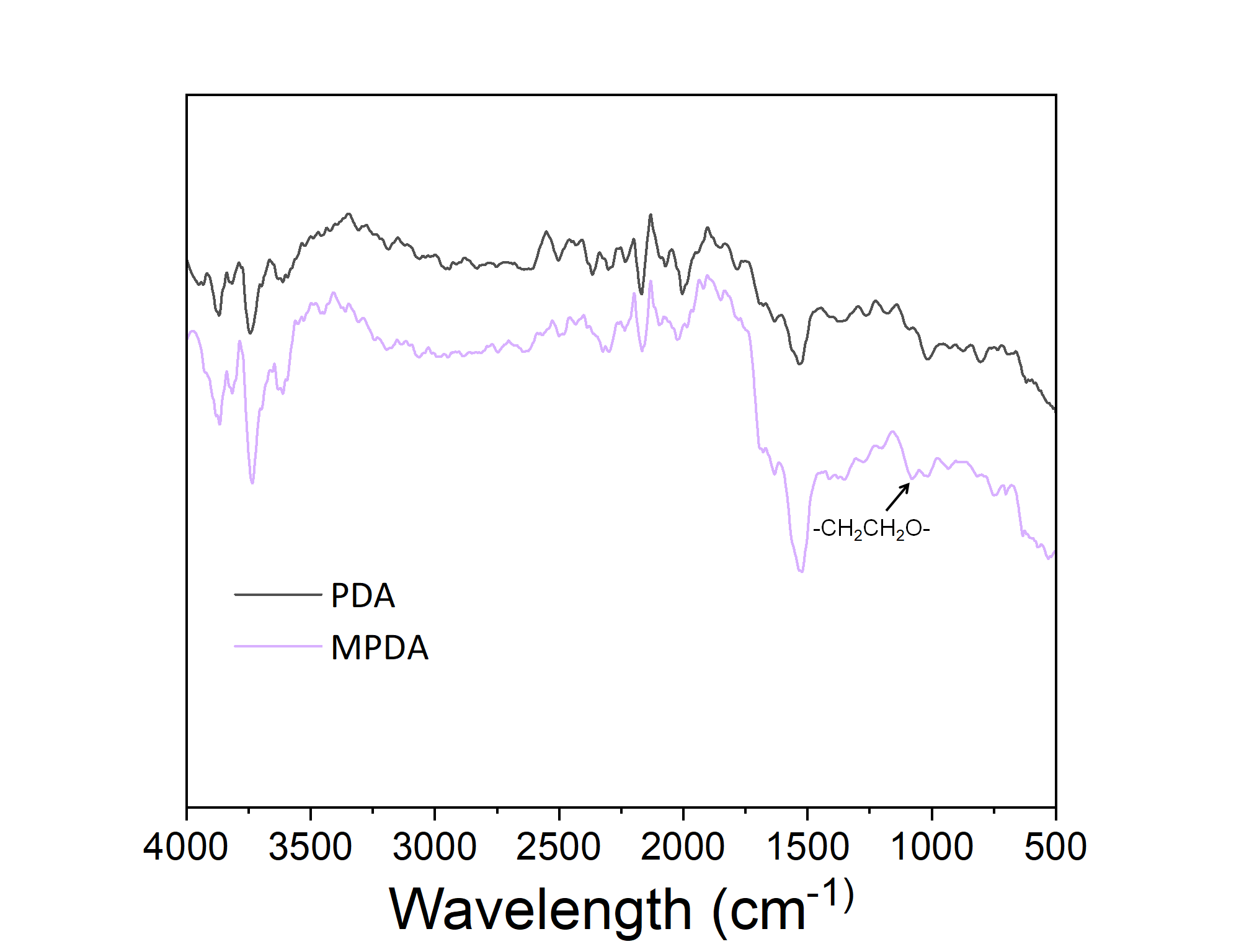

Supplement: Supplementary 1 — Figs. S1 to S16 [file bmr.0019.f1.zip › Fig. S1.tif]

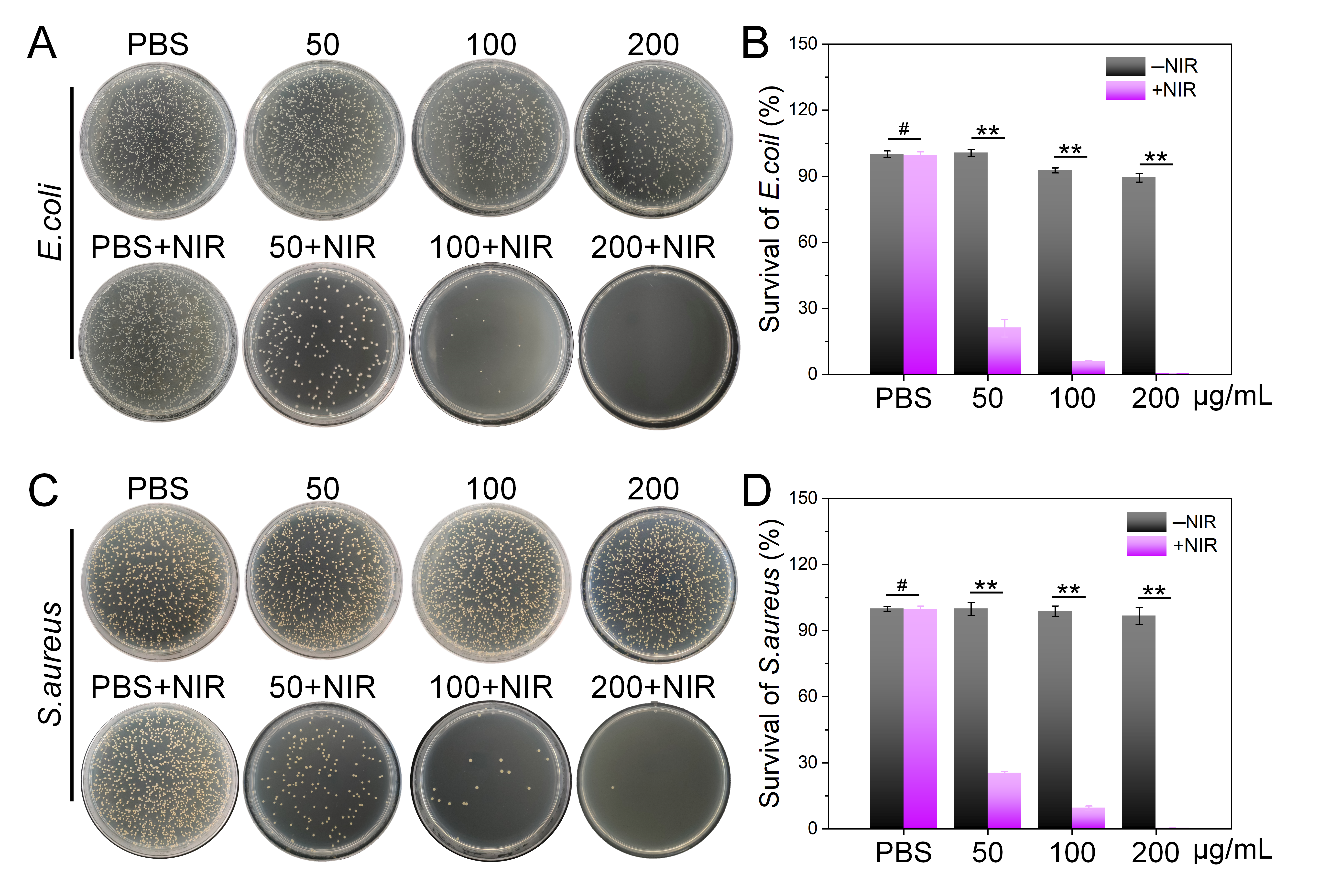

Supplement: Supplementary 1 — Figs. S1 to S16 [file bmr.0019.f1.zip › Fig. S10.tif]

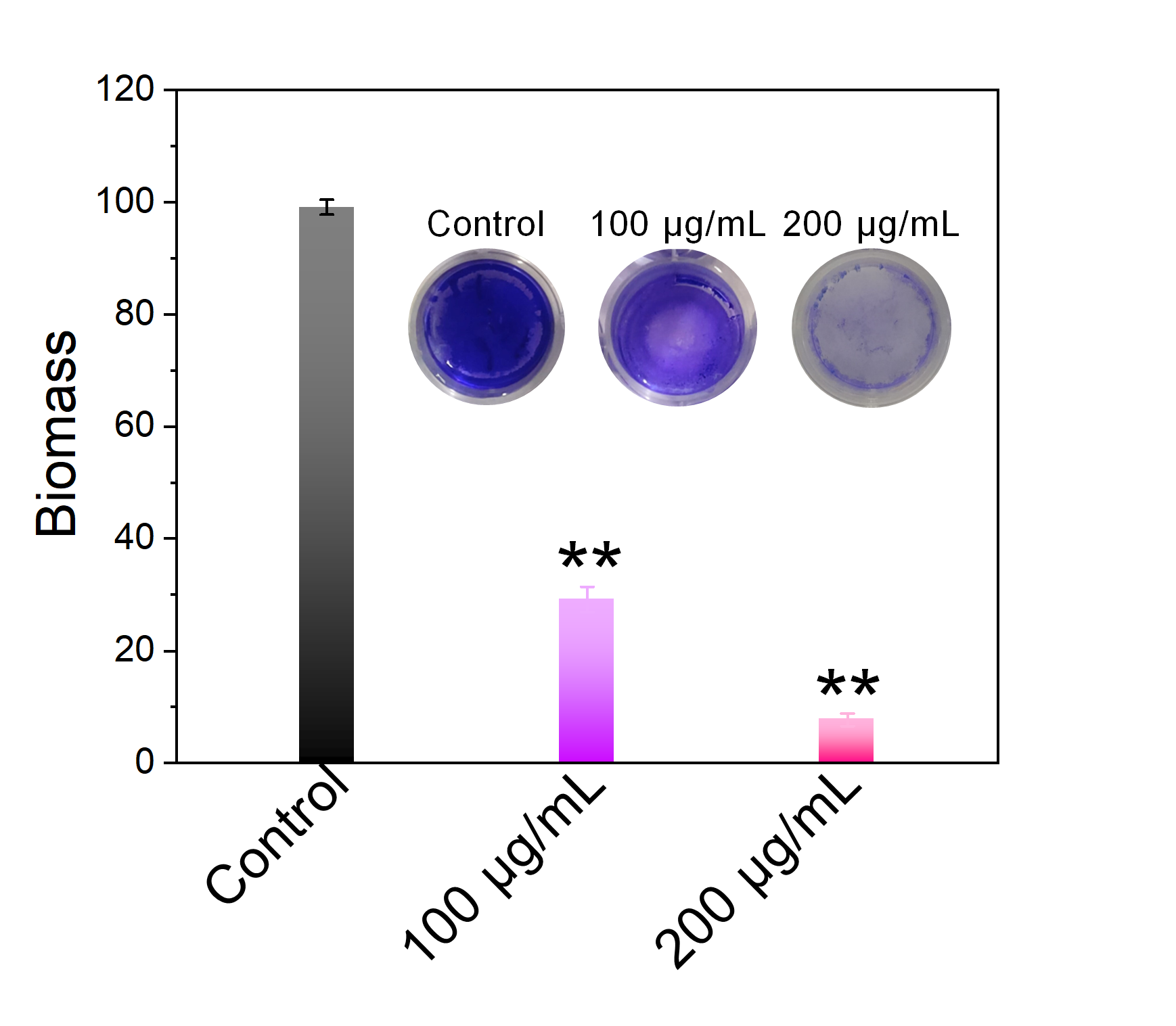

Supplement: Supplementary 1 — Figs. S1 to S16 [file bmr.0019.f1.zip › Fig. S11.tif]

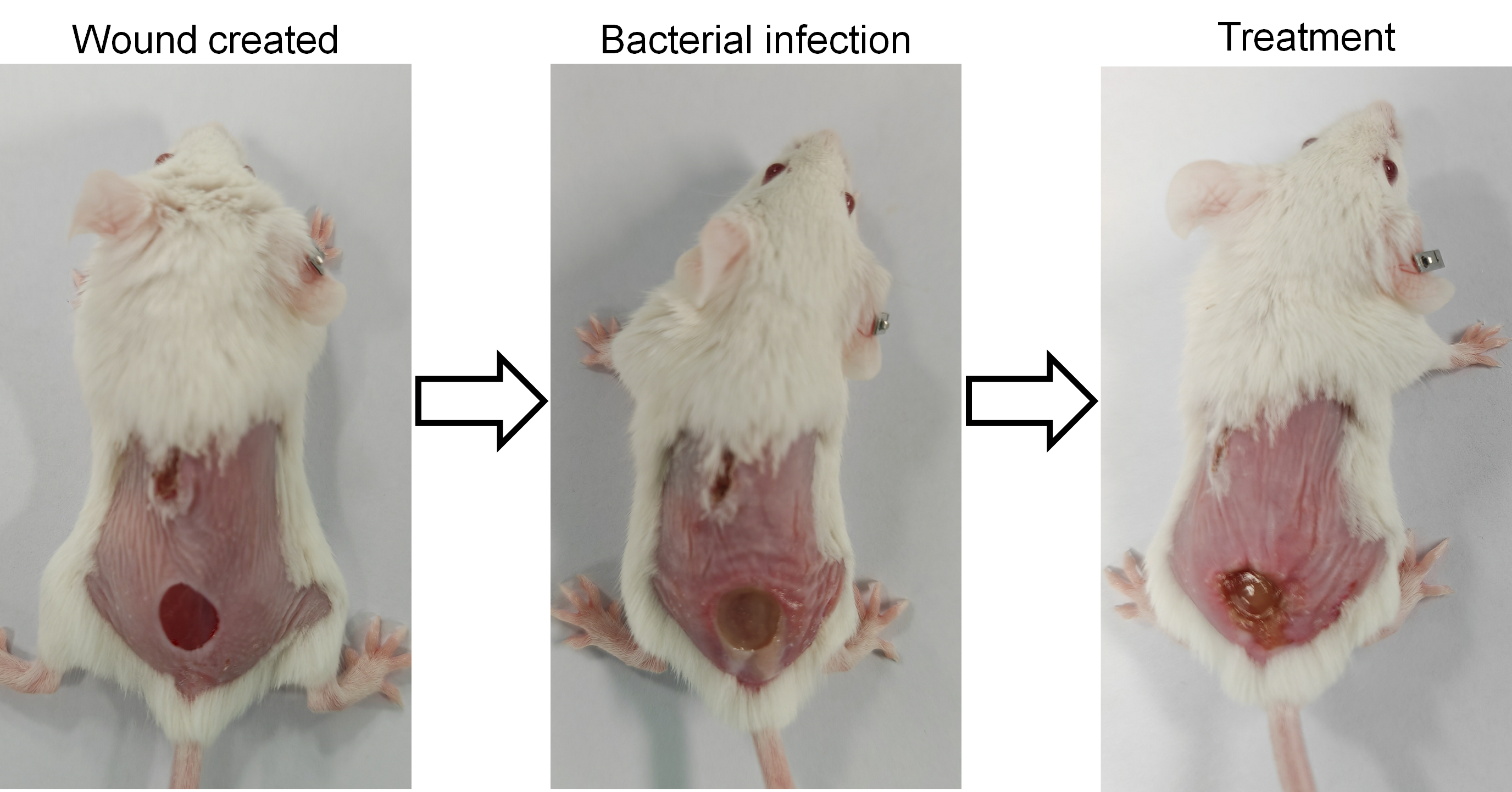

Supplement: Supplementary 1 — Figs. S1 to S16 [file bmr.0019.f1.zip › Fig. S12.tif]

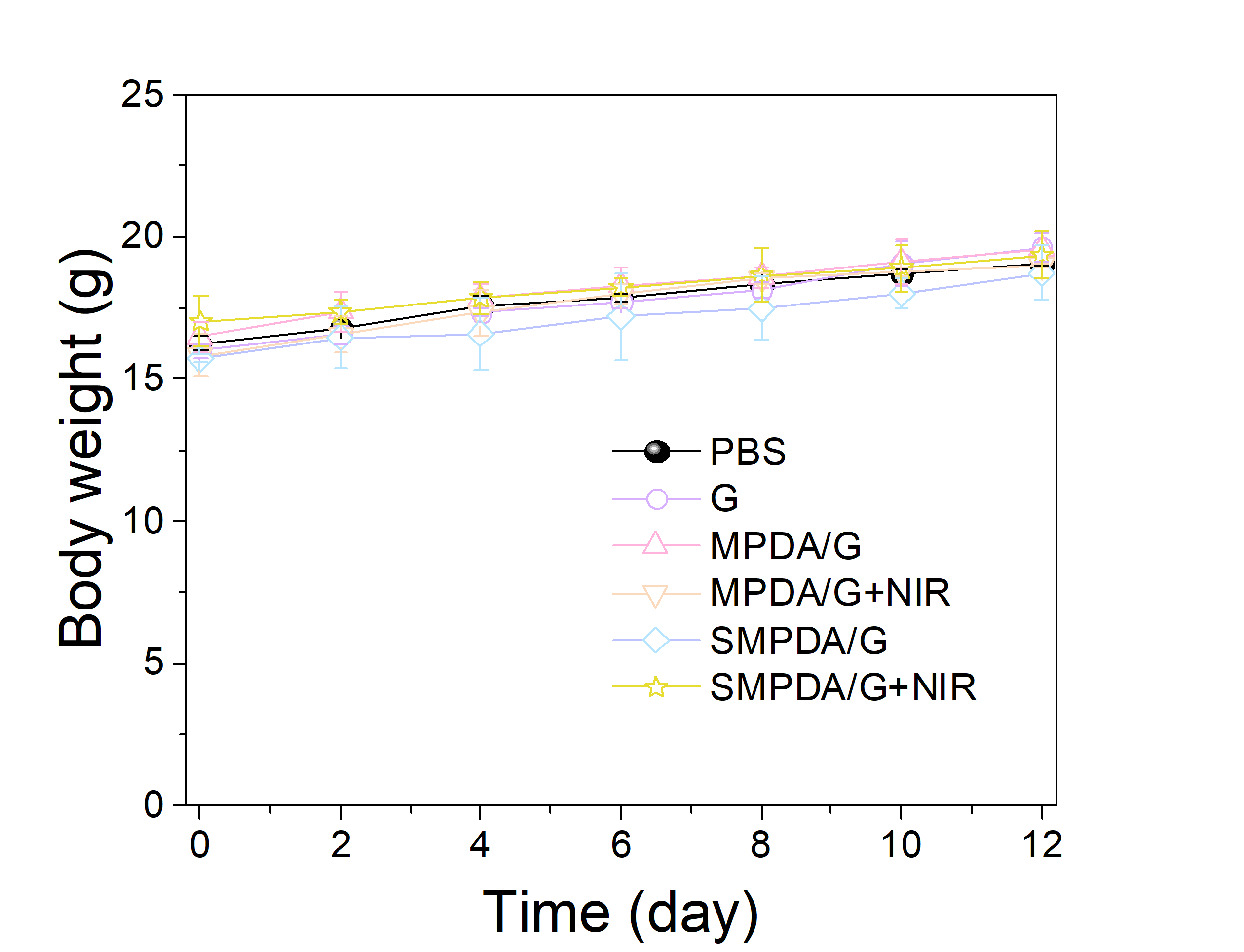

Supplement: Supplementary 1 — Figs. S1 to S16 [file bmr.0019.f1.zip › Fig. S13.tif]

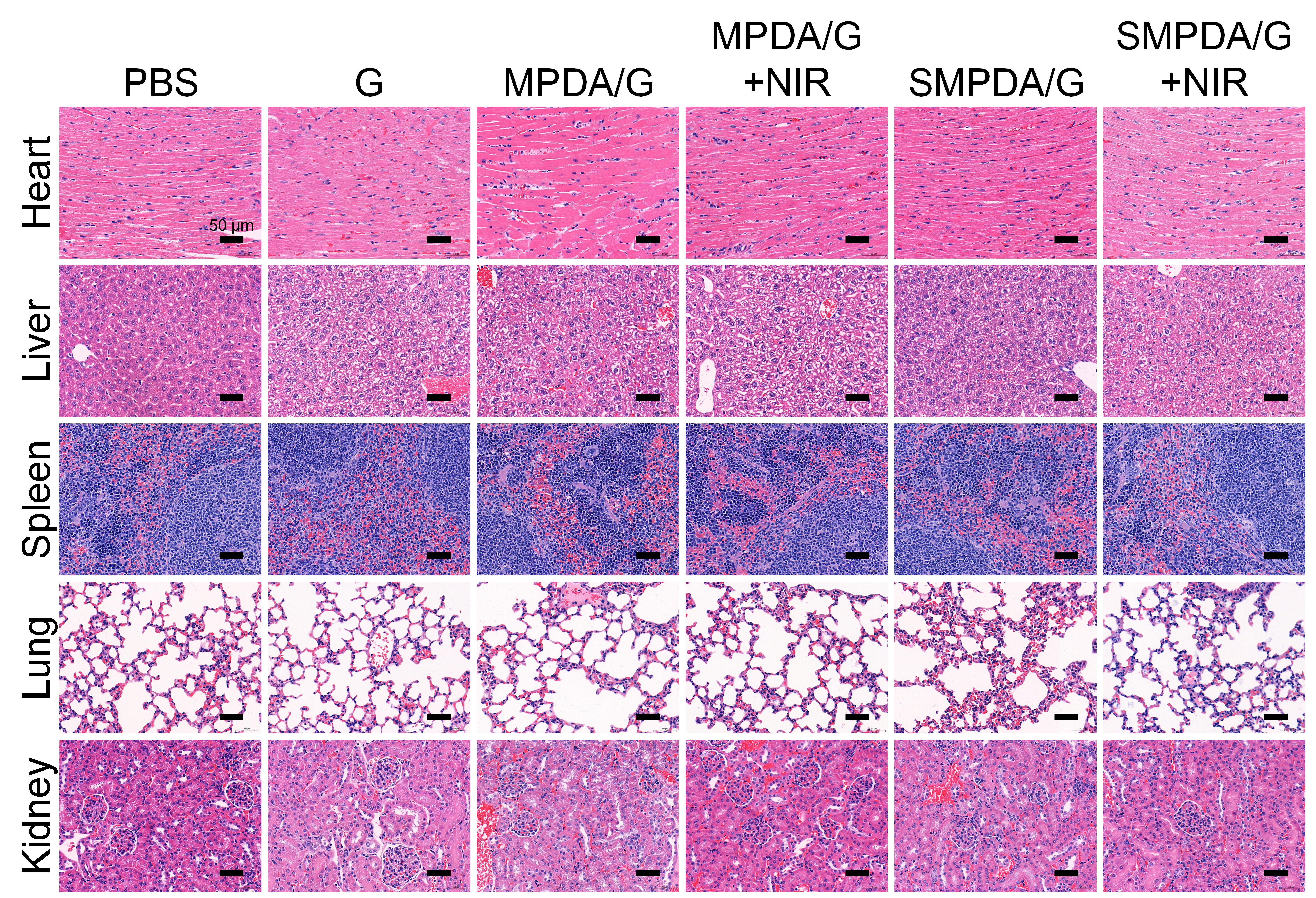

Supplement: Supplementary 1 — Figs. S1 to S16 [file bmr.0019.f1.zip › Fig. S14.tif]

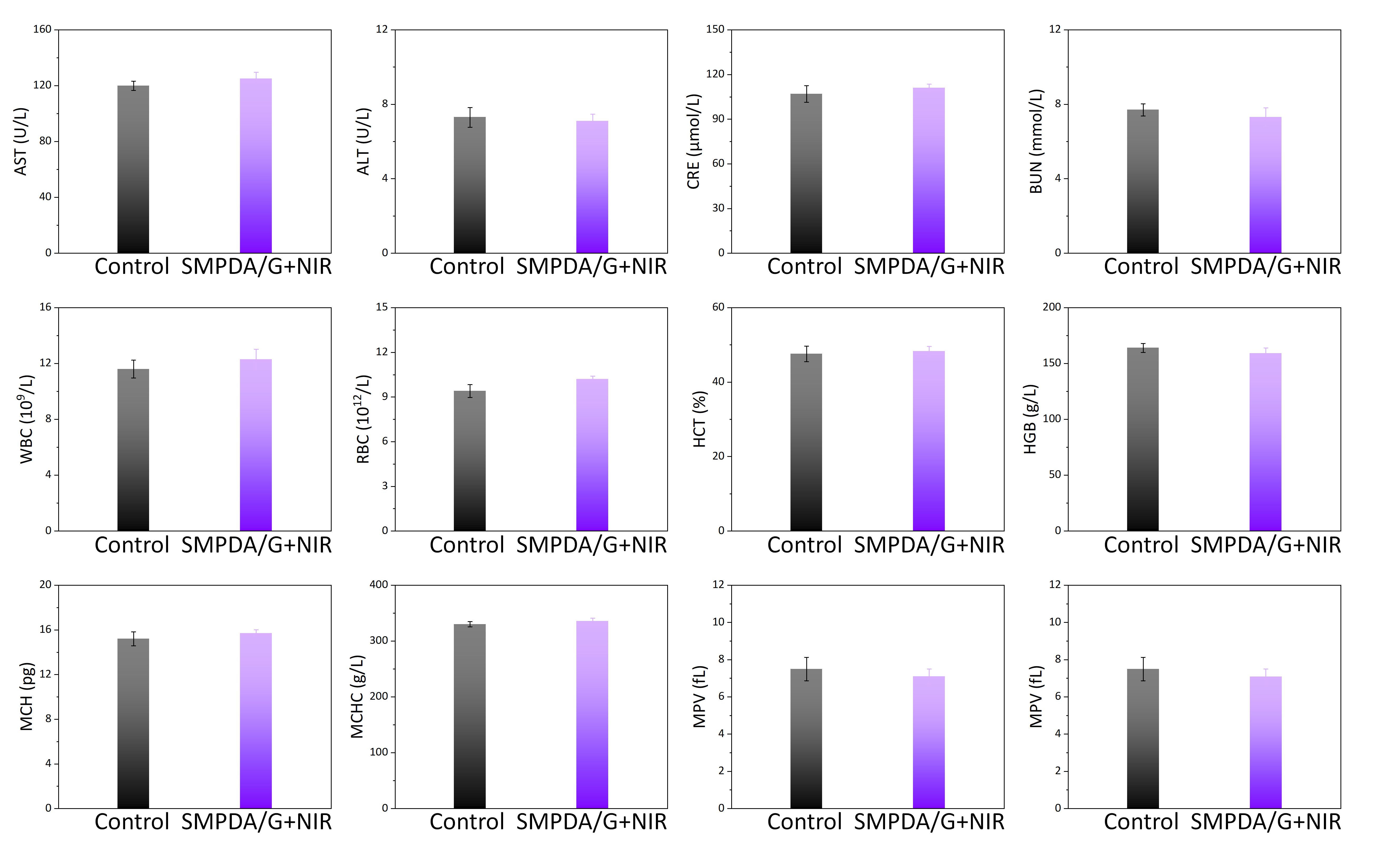

Supplement: Supplementary 1 — Figs. S1 to S16 [file bmr.0019.f1.zip › Fig. S15.tif]

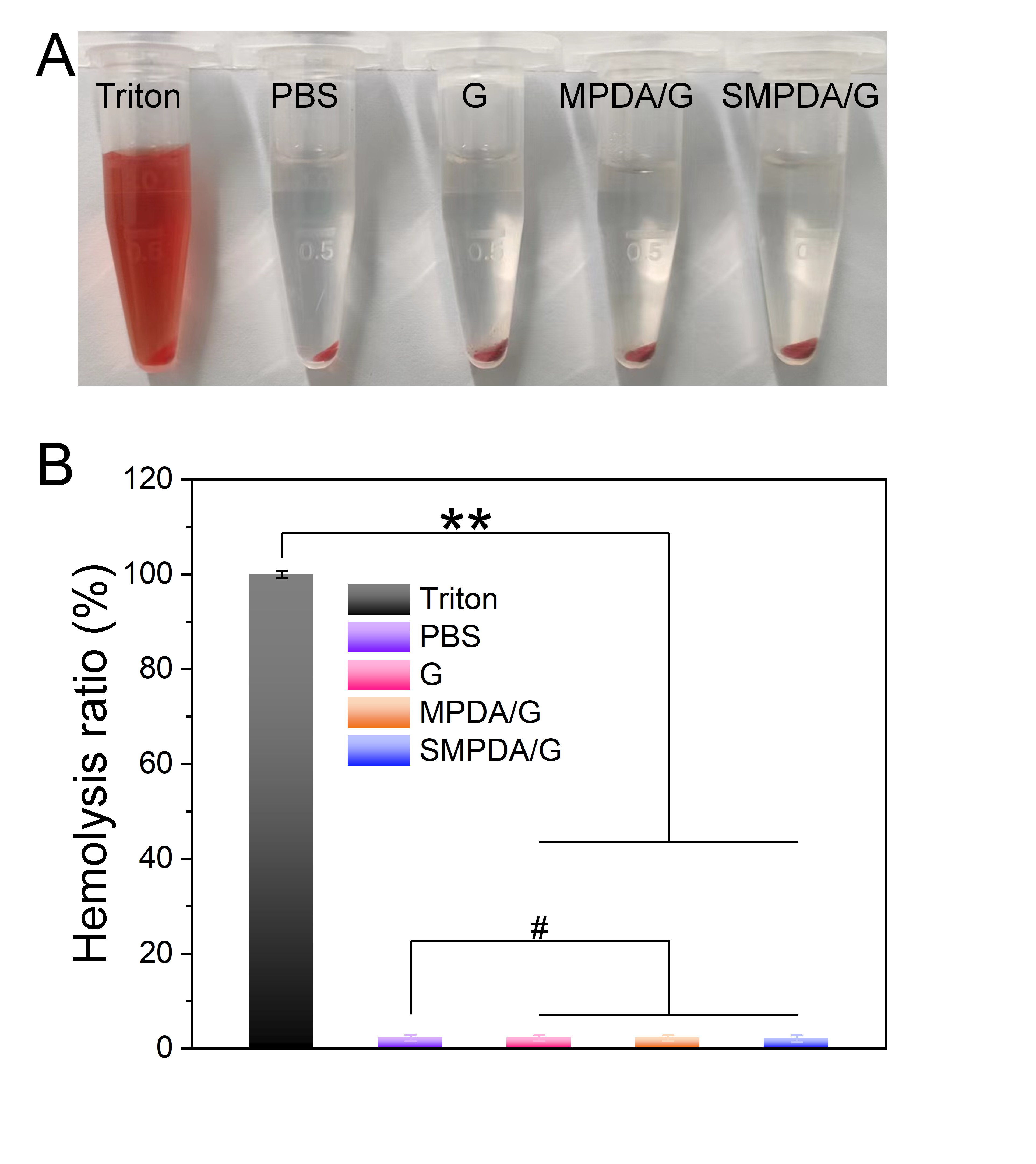

Supplement: Supplementary 1 — Figs. S1 to S16 [file bmr.0019.f1.zip › Fig. S16.tif]

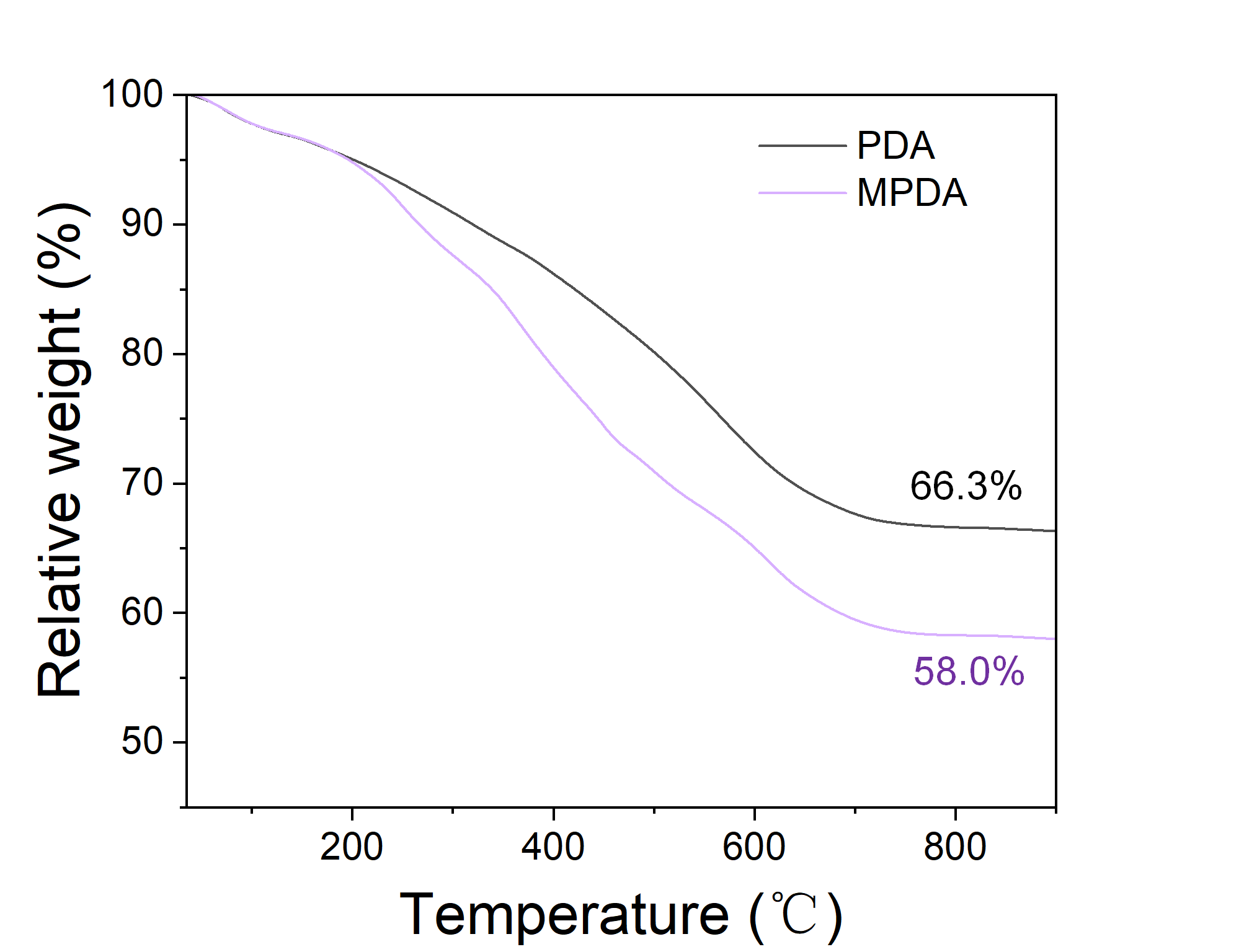

Supplement: Supplementary 1 — Figs. S1 to S16 [file bmr.0019.f1.zip › Fig. S2.tif]

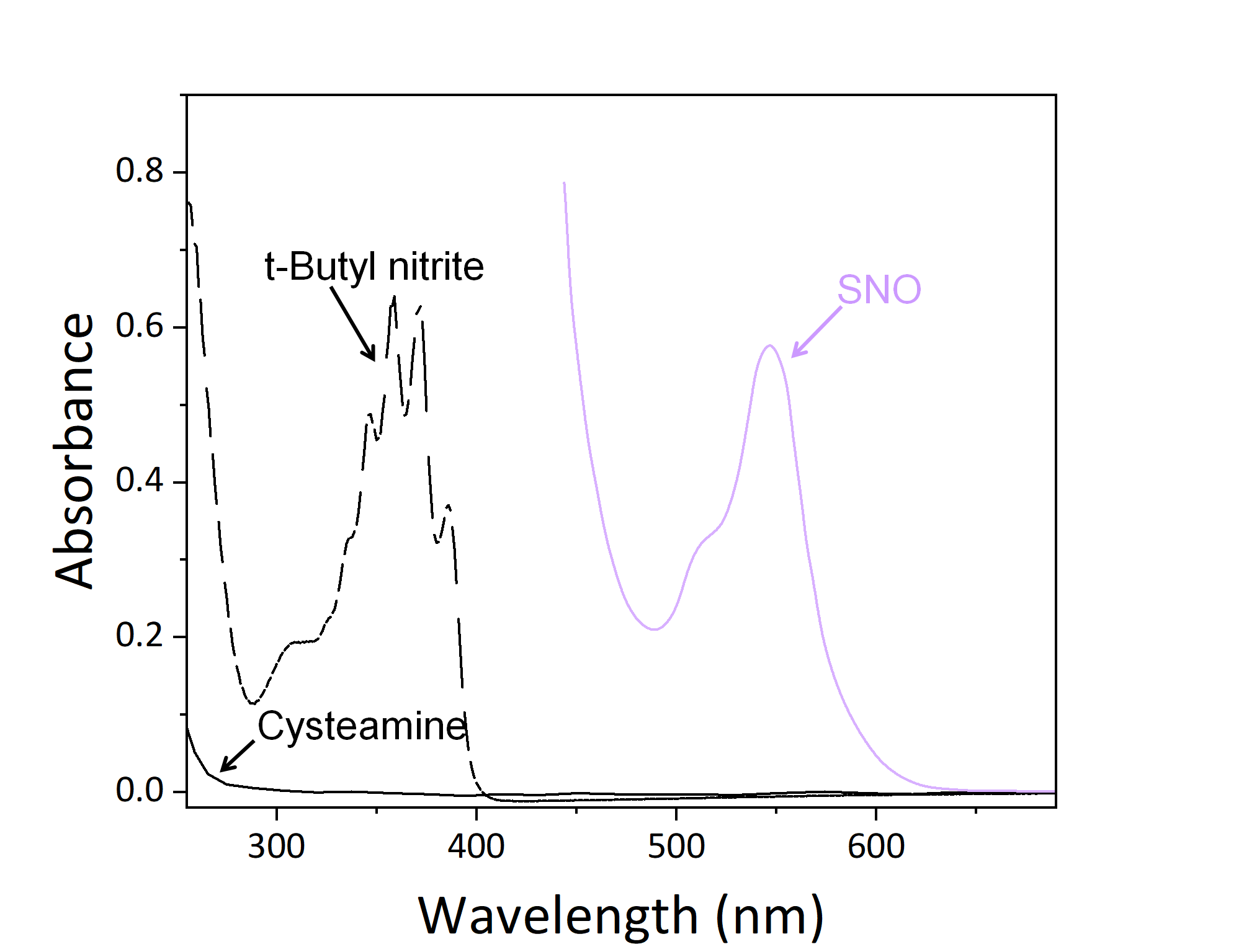

Supplement: Supplementary 1 — Figs. S1 to S16 [file bmr.0019.f1.zip › Fig. S3.tif]

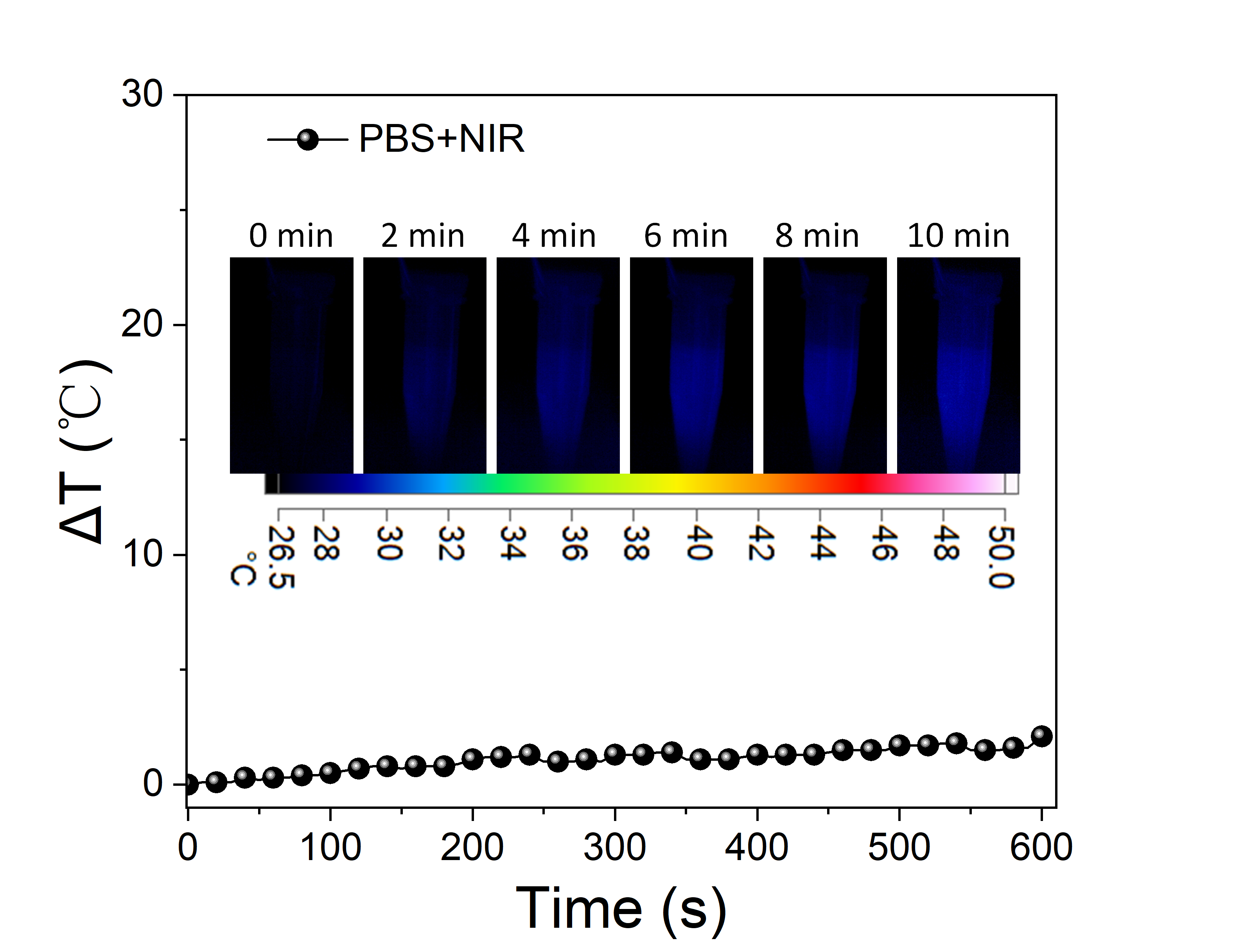

Supplement: Supplementary 1 — Figs. S1 to S16 [file bmr.0019.f1.zip › Fig. S4.tif]

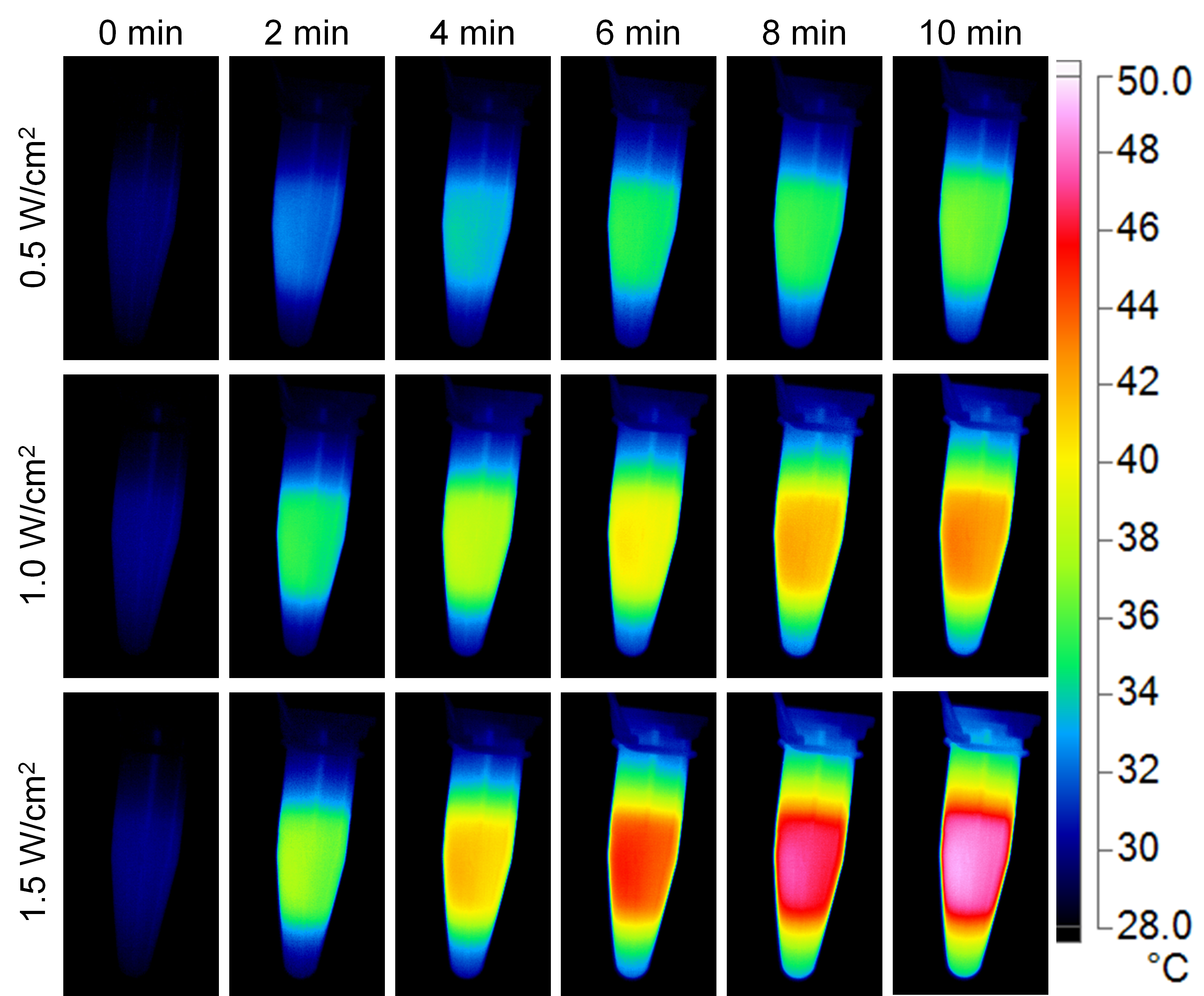

Supplement: Supplementary 1 — Figs. S1 to S16 [file bmr.0019.f1.zip › Fig. S5.tif]

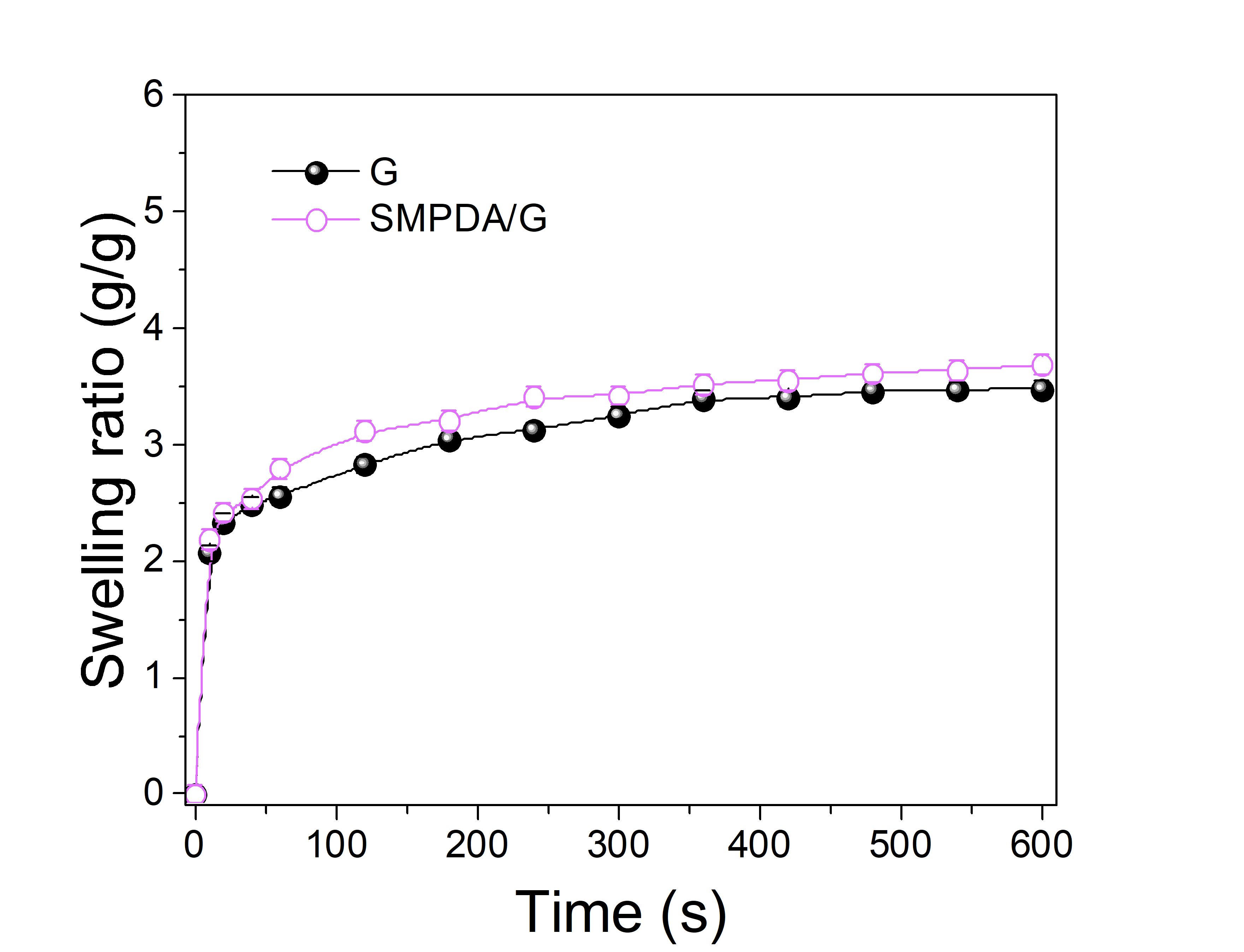

Supplement: Supplementary 1 — Figs. S1 to S16 [file bmr.0019.f1.zip › Fig. S7.tif]

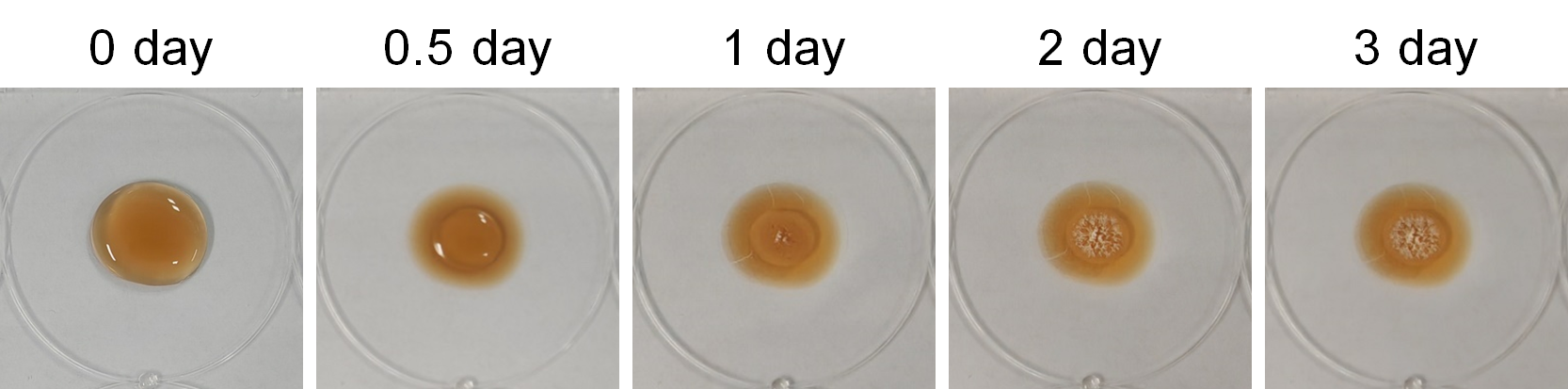

Supplement: Supplementary 1 — Figs. S1 to S16 [file bmr.0019.f1.zip › Fig. S8.tif]

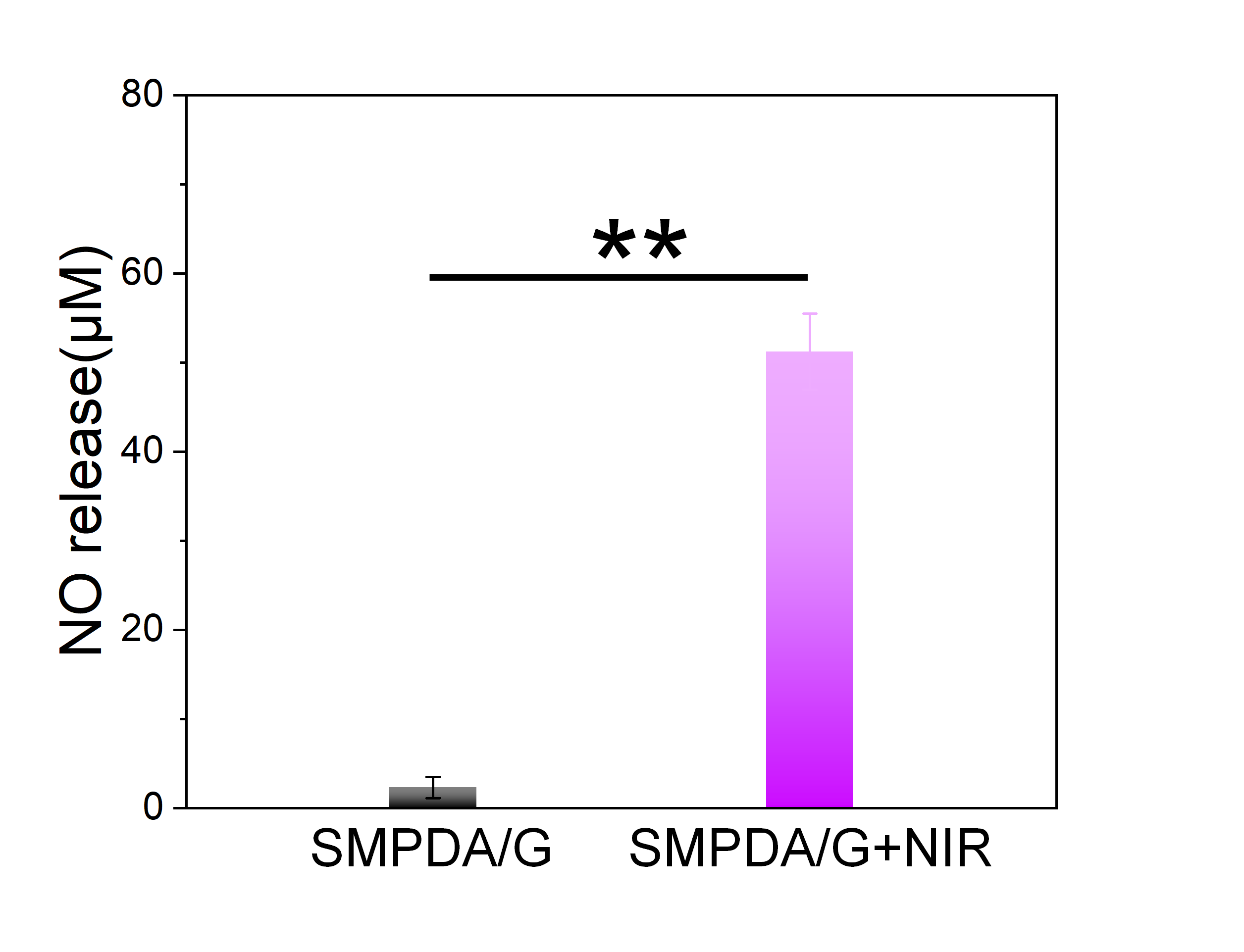

Supplement: Supplementary 1 — Figs. S1 to S16 [file bmr.0019.f1.zip › Fig. S9.tif]
